# Reliability of two different measuring techniques with computer tomography for penetration and distribution of cement in the proximal tibia after total knee arthroplasty

**DOI:** 10.1186/s12891-020-03390-3

**Published:** 2020-06-12

**Authors:** Hennie Verburg, Linda van Zeeland Koobs, Dieu Donné Niesten, Peter Pilot, Nina Mathijssen

**Affiliations:** 1 Department of Orthopaedic Surgery, Reinier de Graaf Groep, Reinier de Graafweg 5, 2625 AD Delft, The Netherlands; 2 Department of Radiology, Reinier de Graaf Groep, Reinier de Graafweg 5, 2625 AD Delft, The Netherlands; 3 Department of Orthopaedic Research, Reinier de Graaf Groep, Reinier de Graafweg 5, 2625 AD Delft, The Netherlands

**Keywords:** Computer tomography, Total knee Arthroplasty, Cement distribution, Cement penetration, CT scan, TKA

## Abstract

**Background:**

To evaluate the reliability of two different techniques for measuring penetration and distribution of the cement mantle in the proximal tibia after total knee arthroplasty (TKA) with Computer Tomography (CT) in vivo.

**Methods:**

Standardized CT scans of the proximal tibia were taken 1 to 2 years after total knee arthroplasties implanted with a surface cementing technique. These prospectively acquired transversal CT images of the surface of the proximal tibia were divided into four quadrants and were assessed once manually and once with a numerical computing program (MATLAB® Update 2, The MathWorks, Inc.) based on Hounsfield Units by one of the researchers. The assessments were repeated by the same and a second researcher. The ratio cement/trabecular bone was calculated 1, 3 and 5 mm distal of the tibia tray per quadrant. Kruskall-Wallis tests with multiple pairwise comparisons (Dunn’s test) were used to determine differences between the quadrants. Intra- and inter-rater reliability as well as the inter method reliability were assessed with the Intraclass Correlation Coefficient (ICC) per level of depth and with Bland-Altman plots.

**Results:**

A total of 92 CT scans were included. The intra- and inter-rater reliability of the manual method ranged from 0.22 and 0.52. The intra- and inter-rater reliability of the matlab method varied between 0.98 to 0.99.

The median percentage cement measured with the matlab method 1 mm underneath the tibial tray varied between 82 and 88%; at 3 mm depth between 38 and 54% and at 5 mm between 15 and 25%. There was significantly (*p* < 0.05) less cement in the antero-medial quadrant compared to the antero-lateral and postero-lateral quadrant at 3 mm and 5 mm depth.

**Conclusions:**

Distribution and penetration of cement in the proximal tibia in a total knee arthroplasty can be measured reliably with CT in combination with the matlab method presented in this manuscript. This method can be used for clinical purposes as well as for scientific research.

**Trial registration:**

METC-nr: 06–104 Dossier NL14807.098.06/versie 06.

## Background

Total Knee Arthroplasty (TKA) is a very successful orthopedic procedure. Numerous studies have confirmed excellent results, with 10 to 15 year survivorship of more than 90% [1–4]. Nevertheless, there are still patients with complaints, and in a large number of cases these complaints are due to aseptic loosening of the prosthesis [5, 6]. Aseptic loosening occurs mostly at the tibia component and might be caused by suboptimal fixation of the prosthesis. Knee prostheses fixated with bone cement (Polymethylmethacrylaat = PMMA) have equally good or even better results regarding aseptic loosening and clinical outcome than knee prostheses fixated without bone cement [7, 8]. The key to optimizing the interfacial strength is achieving and maintaining maximal infiltration of cement into the bone to obtain large inter-digitation and a large contact area [9, 10].

Several studies concluded that optimal fixation requires penetration of cement into the proximal tibia of 3 to 4 mm [10–12]. By contrast, recent in vitro research stated that a mean penetration of 1.1 mm is sufficient and that it is not necessary to achieve a penetration of more than 2 mm to have a good pull-out strength [13]. In vivo, however, the tibial tray is not only subject to pull-out forces but also and may be even more so to compression and shearing forces. The volume of cement penetrating into the bone is less predictive of failure than the mean penetration, which indicates the importance of a good distribution of the cement [13].

So far, there is no good method for evaluating the penetration and distribution of the cement in vivo. Until now, the penetration depth of the cement is measured on conventional radiographs with a ruler. This is a rough method and it only allows measurements in the coronal or sagittal plane and not in the transverse plane. The distribution of the cement cannot be visualized with conventional radiography, nor is it possible to obtain a three-dimensional image with this technique. By contrast, in a cadaver model, Computer Tomography (CT) proved to be a reliable procedure to assess the cement mantle in the proximal tibia, with the metal prosthesis in situ [14, 15]. So far, however, this procedure has not been evaluated in vivo. To our knowledge, no clinical studies about the validation of CT-based cement penetration depth and distribution assessment in TKA have been published yet.

We hypothesized that Computer Tomography (CT) is a reliable and adequate method for in vivo assessment of the penetration and distribution of the cement in the proximal tibia after cementing a total knee arthroplasty. The secondary aim was to study if assessment of the cement with a numerical computing method is just as reliable as a manual method. Furthermore, the third aim of the study was to study the cement penetration and cement distribution underneath the tibial tray in vivo*.*

## Methods

**Fig. 1:**
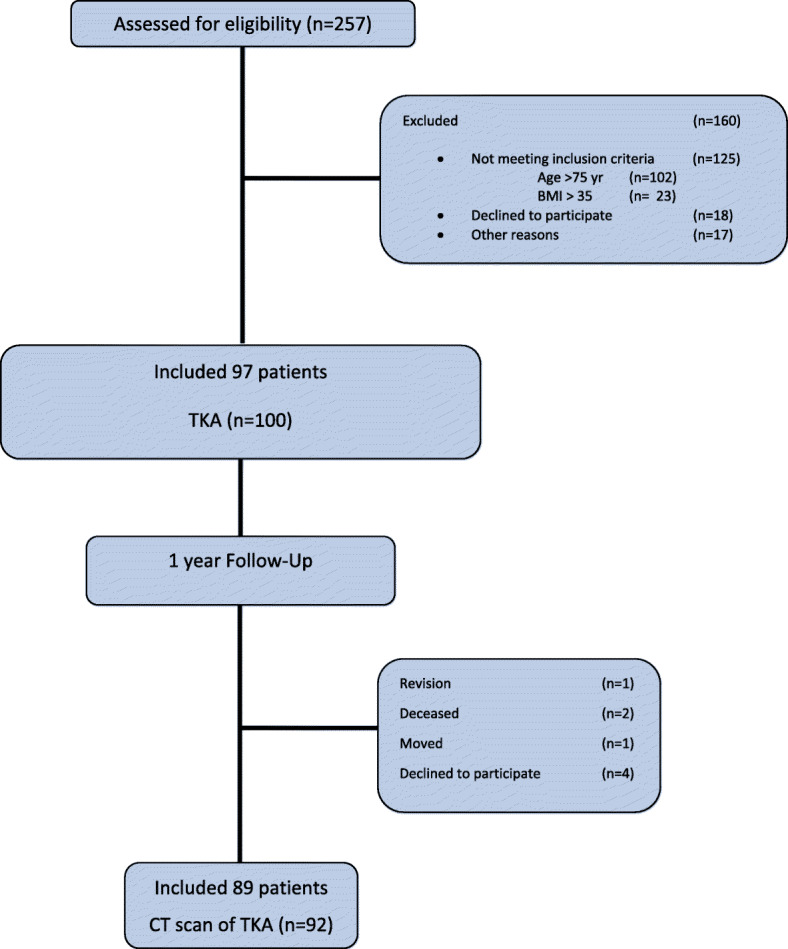
Flow diagram of included patients. BMI = body mass index, TKA = total knee arthroplasty

Two hundred fifty seven patients were initially asked, prior to surgery, to participate in a prospective clinical study that evaluated the clinical and radiographic results of two different approaches for primary TKA, the mini mid-vastus and the conventional approach [16]. In total 97 patients (100 TKA) were included in that clinical study. Part of the radiographic follow-up of that clinical study was a CT scan 1 to 2 years post-operative. For this CT scan study, we included 92 CT scans (Fig. 1). We used these CT scans in this study for assessing the cement mantle of the tibial tray of the total knee prosthesis.

Inclusion criteria of the prospective clinical study were patients from 18 to 75 years of age with a Body Mass Index (BMI) of less than 35 kg/m^2^ who were diagnosed with osteoarthritis and indicated for a primary TKA. Exclusion criteria were the need of another joint replacement within 6 months, severe varus or valgus malalignment (> 15 degrees), extreme pre-existing knee deformities, previous knee surgery except arthroscopy, and unwillingness to cooperate in this study.

We started this CT scan study between 1 and 2 years after patients underwent surgery for primary TKA in the prospective clinical study. The clinical and the CT scan study were both approved by the local Medical Ethics Committee (NL14807.098.06) and all patients gave written consent.

### Surgery

**Fig. 2:**
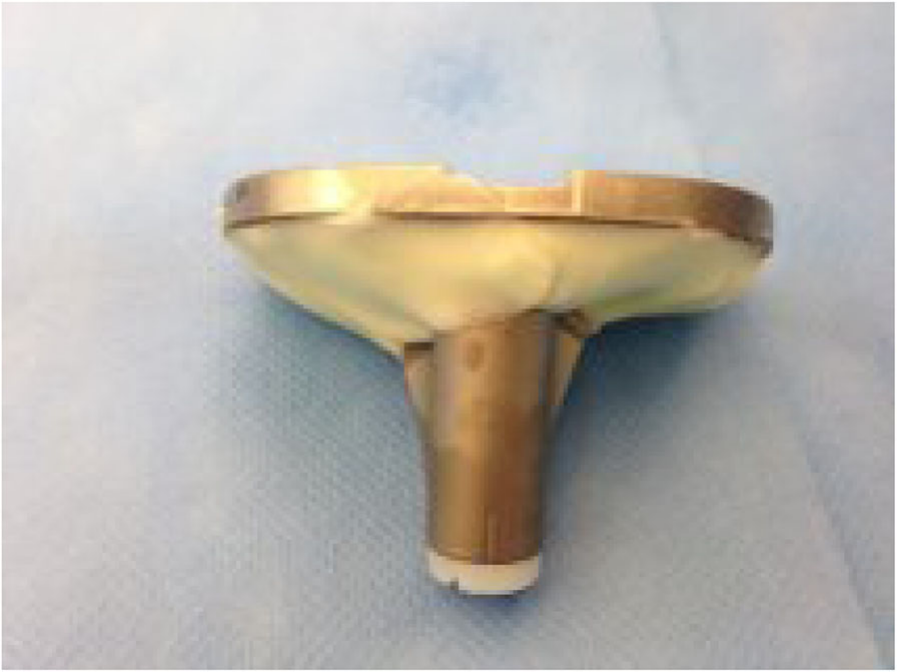
Tibial tray just before introducing into the proximal tibia with cement applied by surface cementing technique

The surgeries were performed by two experienced orthopedic knee surgeons. All patients were operated under general or spinal anesthesia. Antibiotics were administered for 24 h and a dynamic leg holder and a tourniquet were used. All patients received the NexGen® LPS- High Flex Fixed Bearing Knee (Zimmer Biomet®, Warsaw, IN, USA). A surface cementing technique was applied using Palacos R + G® bone cement (Zimmer Biomet®, Warsaw, IN, USA). In this technique, the cement is applied at the undersurface of the tibial tray only, and the stem is placed press fit to achieve fixation (see Fig. 2).

### Computer tomography

Standardized CT scans were performed 1 to 2 years post-operatively with a Toshiba Aquilion® 32 slice scanner. The CT scanner was set at 135 kV, 150 mA and a slice thickness of 0.5 mm using an osseous mixed filter. Reconstructions were made with a helical pitch 21 and image thickness reconstruction of 2 mm. To do all measurements in the same way the CT images were re-sampled perfectly parallel to the tibia baseplate.

### Data analysis

**Fig. 3:**
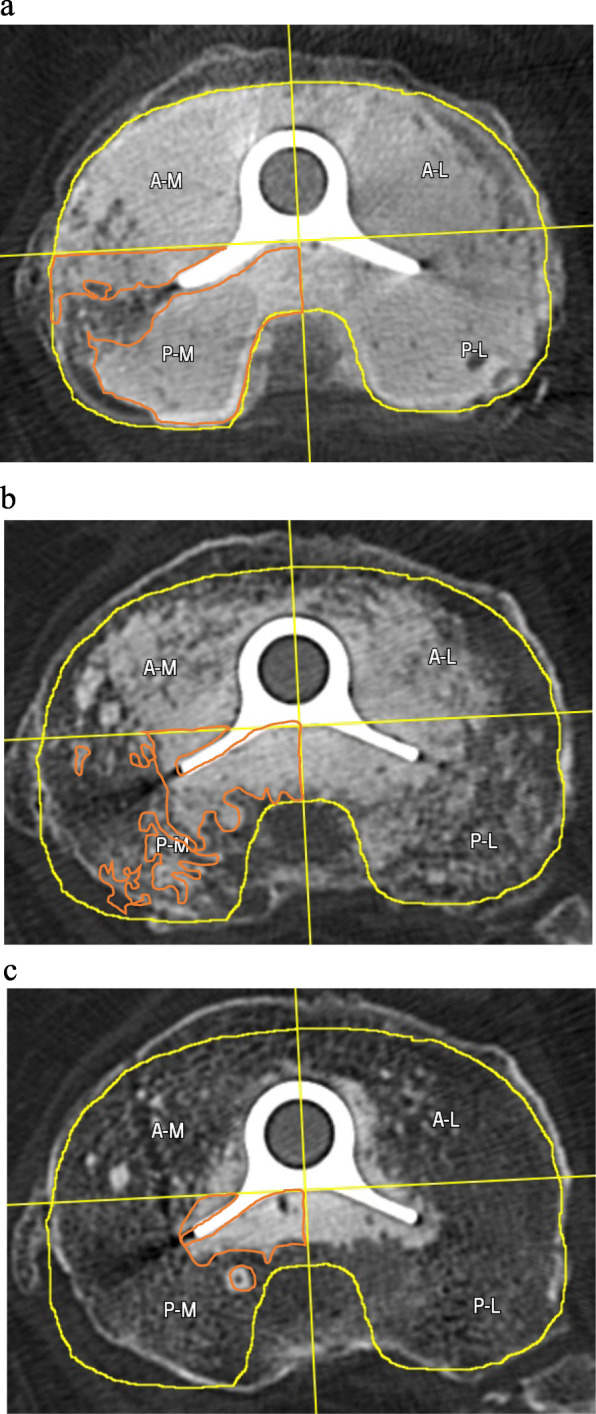
**a** CT scan 1 mm below the tibial tray. There is an equal distribution of the cement. The yellow line is the projection of the circumference of the tibial tray. The orange line is the circumference of the area with cemented trabecular bone in the Postero-Medial quadrant. A-M = Antero-Medial, A-L = Antero-Lateral, P-M = Postero-Medial, P-L = Postero-Lateral. **b** CT scan 3 mm below the tibial tray. There is less cement in the periphery of all the three quarters than in the CT slice 1 mm below the tibial tray (Fig. 3a). The yellow line is the projection of the circumference of the tibial tray. The orange line is the circumference of the area with cemented trabecular bone in the Postero-Medial quadrant. A-M = Antero-Medial, A-L = Antero-Lateral, P-M = Postero-Medial, P-L = Postero-Lateral. **c** CT scan 5 mm below the tibial tray. There is overall less cement in the trabecular bone than in the CT slices 1 mm and 3 mm below the tibial tray. There is less cement in the anterior quadrants than in the posterior quadrants. The yellow line is the projection of the circumference of the tibial tray. The orange line is the circumference of the area with cemented trabecular bone in the Postero-Medial quadrant. A-M = Antero-Medial, A-L = Antero-Lateral, P-M = Postero-Medial, P-L = Postero-Lateral

The CT reconstructions were assessed using the graphics module CARESTREAM Vue PACS (Picture Archiving and Communication System, Carestream.com). This PACS graphics module was used to draw the outlines of the Range of Interest by the researchers (see for illustration the orange line in Fig. 3). The assessments were done in the CT-slices 1, 3 and 5 mm below the slice with the last visual metal from the tibial baseplate.

After drawing purely visual and with manual contouring (manual method) the different Ranges of Interest (prosthesis, trabecular bone and cemented trabecular bone), the PACS graphics module measured these areas in square millimeters.

Also, the CT reconstructions were assessed with a numerical computing program (MATLAB® Update 2, The MathWorks, Inc.) based on Hounsfield Units (HU) (matlab method, see additional files for the matlab scripts). The HU was set between 400 and 550 HU for trabecular bone, between 550 and 1600 HU for cement or cemented trabecular bone, 1600–2110 for cortical bone and > 2100 HU for prosthesis [14, 15].

The area of the proximal tibia was divided into 4 quadrants, the antero-medial (A-M), the antero-lateral (A-L), the postero-medial (P-M) and the postero-lateral (P-L) quadrant to examine the distribution of the cement in those quadrants in particular for all levels of dept.

The matlab method subtracted the area of the prosthesis and the area of cortical bone from the total area. The area of cemented and uncemented trabecular bone was defined as 100%. Of this surface we calculated the areas of trabecular bone with or without cement as a part of it in percentages. In this way, we could examine the cement penetration per quadrant into the trabecular bone of the proximal tibia at a depth of 1, 3 and 5 mm in percentages.

The measurements, both the manual method and the matlab method, were done by one experienced researcher. The first 50 consecutive measurements were repeated by the same researcher, who was blinded with regard to the first assessments and also by a second researcher.

### Statistics

Analyses were performed using SPSS® Statistics for Windows Version 21, Chicago, IL, USA. Intra- and inter-rater reliability as well as the inter-method reliability were assessed with the Intraclass Correlation Coefficient (ICC) per level of depth and with Bland-Altman plots. Kruskal-Wallis tests with multiple pairwise comparisons (Dunn’s test) were used to determine differences between the quadrants. Significance values for the pairwise comparisons were adjusted by the Bonferroni correction for multiple tests. The level of significance was set at *p* < 0.05.

## Results

From August 2007 to June 2009, 257 patients were assessed for eligibility, 97 of whom were included in the prospective clinical study (see Fig. 1). Four of the included patients could not be included in the CT scan study: 2 patients had died from cancer, 1 patient moved abroad and in 1 patient the TKA had been revised for persistent pain below the medial tibia plateau. Consequently, 93 patients were asked to participate in the CT scan study, 3 of whom had a bilateral TKA, and 89 (92 TKAs) gave informed consent. Of these patients, 66% (59 patients) were female. Mean age was 67 years (SD 6.6), and the mean BMI was 28.6 (SD 3.2).

**Fig. 4:**
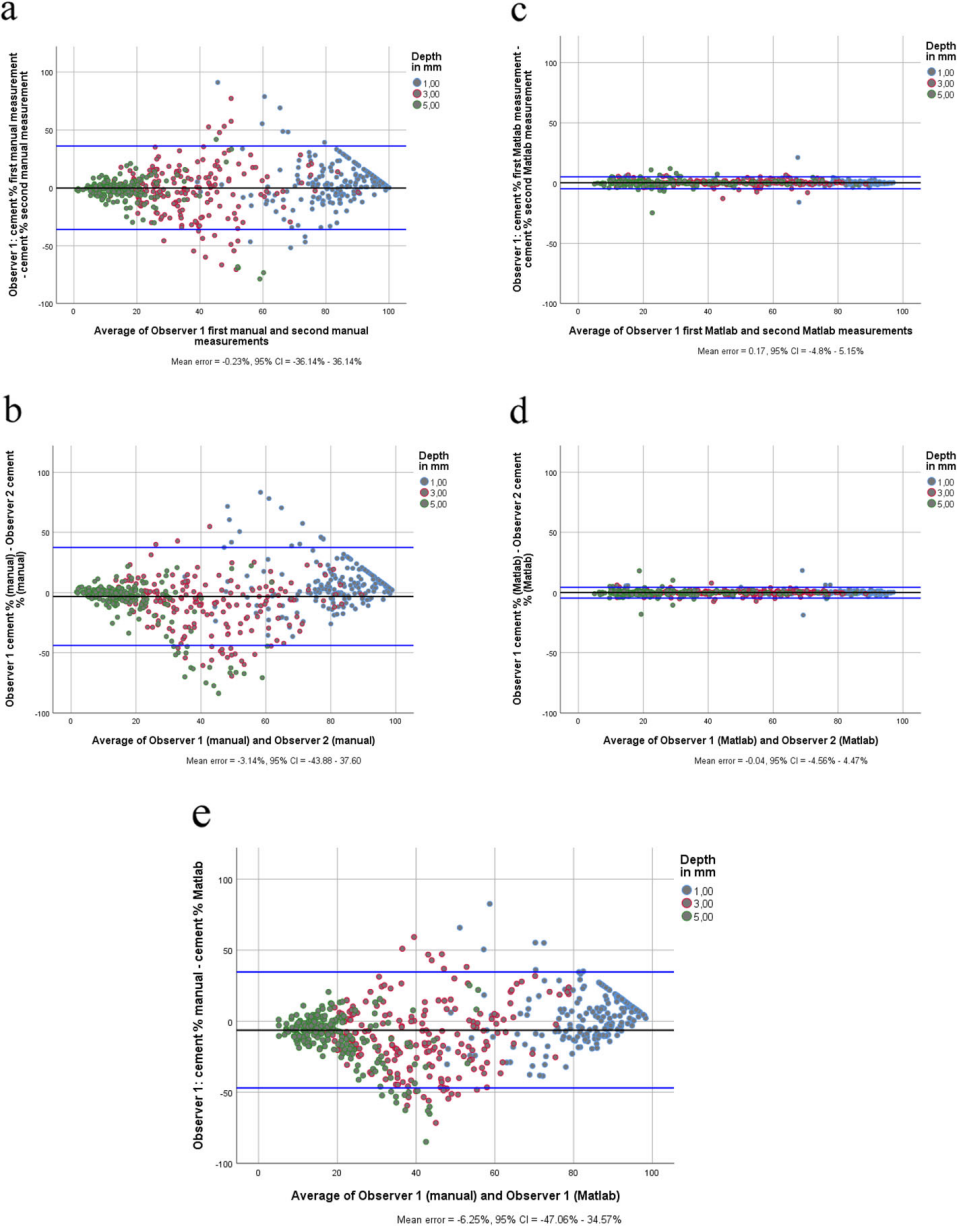
**a** CI = Confidence Interval. **b** CI = Confidence Interval. **c** CI = Confidence Interval. **d** CI = Confidence Interval. **e** CI = Confidence Interval

The intra- and inter-rater variability of the manual method ranged from 0.22 and 0.52 for the various depths. The intra- and inter-rater reliability of the matlab-method varied between 0.98 to 0.99 and the inter-method reliability ranged between 0.19 and 0.24 (Table 1). Bland-Altman plots of both methods as well as of the inter-method reliability are shown in Fig. 4. Since the reliability of the matlab method was much better than the manual method we performed the statistical analysis with the results of the matlab method.

**Table 1:** **Table 1** The intra-rater reliability and inter-rater reliability of the manual and the matlab method

	Manual: Intra-rater reliability^a^ ICC (95% CI)	Manual: Inter-rater reliability^b^ ICC (95% CI)	Inter-method reliability^a^ ICC (95% CI)	Matlab: Intra-rater reliability^a^ ICC (95% CI)	Matlab: Inter-rater reliability^b^ ICC (95% CI)
Cement % at 1 mm	0.26 (0.13–0.39)	0.36 (0.23–0.47)	0.24 (0.11–0.37)	0.99 (0.98–0.99)	0.99 (0.98–0.99)
Cement % at 3 mm	0.35 (0.22–0.47)	0.52 (0.42–0.62)	0.22 (0.09–0.35)	0.99 (0.99–0.99)	0.99 (0.99–0.99)
Cement % at 5 mm	0.37 (0.25–0.49)	0.22 (0.08–0.35)	0.19 (0.06–0.32)	0.98 (0.98–0.98)	0.99 (0.98–0.99)

^a^Observer 1

^b^Observer 1 (first measurement) vs. Observer 2

ICC = Intraclass Correlation Coefficient

*CI* Confidence Interval

**Fig. 5:**
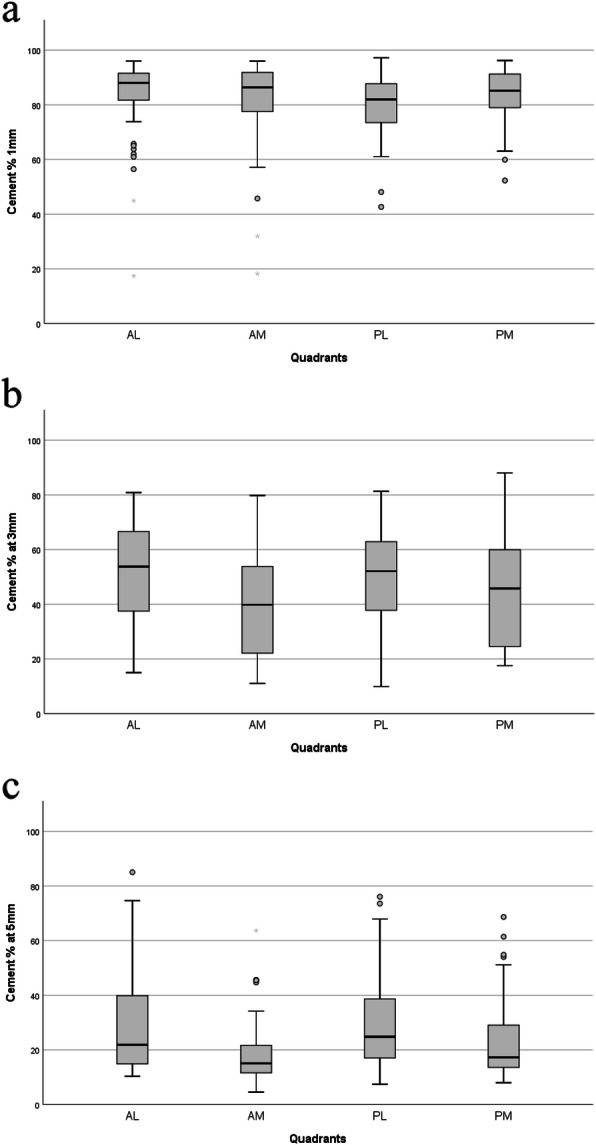
**a**: Measurements with the Matlab Method. Cement distribution 1 mm underneath the tibial tray over the 4 quadrants. AL = Antero-Lateral quadrant, AM = Antero-Medial quadrant, PL = Postero-Lateral quadrant, PM = Postero-Medial quadrant. **b**: Measurements with the Matlab Method. Cement distribution 3 mm underneath the tibial tray over the 4 quadrants. AL = Antero-Lateral quadrant, AM = Antero-Medial quadrant, PL = Postero-Lateral quadrant, PM = Postero-Medial quadrant. **c**: Measurements with the Matlab Method. Cement distribution 5 mm underneath the tibial tray over the 4 quadrants. AL = Antero-Lateral quadrant, AM = Antero-Medial quadrant, PL = Postero-Lateral quadrant, PM = Postero-Medial quadrant

The median percentages cement at 1, 3 and 5 mm beneath the tibial tray are shown in Table 2 and Fig. 5. There was significantly less cement in the antero-medial quadrant compared to the antero-lateral and postero-lateral quadrant at 3 mm and 5 mm depth.

**Table 2:** **Table 2** The median percentages cement at 1, 3 and 5 mm beneath the tibial tray

Distance beneath tibial tray	Quadrant	Observer 1 first measurement % surface cement^a^	Observer 1 second measurement % surface cement^a^	Observer 2% surface cement^a^
		Median [IQR]	Median [IQR]	Median [IQR]
1 mm*	AM	86.37 [80.87–91.56]	86.56 [77.50–91.87]	86.24 [77.03–91.93]
	AL	88.06 [80.87–91.56]	88.08 [80.47–91.42]	88.47 [80.72–91.72]
	PM	85.19 [78.76–91.29]	85.21 [78.09–91.25]	85.25 [78.59–90.84]
	PL	81.95 [73.46–87.73]	82.45 [72.91–88.06]	81.99 [72.50–88.03]
3 mm^#^	AM	39.83 [21.96–53.94]	39.19 [22.72–55.58]	38.41 [22.27–53.34]
	AL	53.75 [36.71–66.84]	53.21 [35.76–68.46]	54.34 [37.13–68.31]
	PM	45.76 [24.17–60.07]	46.08 [25.14–58.30]	44.95 [24.96–60.77]
	PL	52.10 [37.64–63.28]	51.86 [36.58–62.26]	52.84 [36.41–64.14]
5 mm^¥^	AM	15.07 [11.55–21.98]	15.72 [11.17–19.39]	14.94 [10.80–20.85]
	AL	21.86 [14.79–40.07]	22.27 [15.74–39.52]	21.97 [15.89–40.71]
	PM	17.31 [13.44–29.62]	17.49 [13.03–28.50]	17.59 [13.95–27.59]
	PL	24.80 [16.88–39.25]	25.54 [16.99–38.87]	25.49 [17.05–39.54]

*AM* Antero-medial, *AL* Antero-lateral, *PM* Postero-medial, *PL* Postero-lateral

^a^ % Surface cement = percentage of trabecular bone surface that is filled with cement

*Distribution over the quadrants was equal (*p* = 0.076)

#Distribution over the quadrants was not equal (*p* = 0.006). Post hoc analysis with a Bonferroni adjustment revealed differences between AM and PL (*p* = 0.025) and between AM and AL (*p* = 0.012)

^¥^Distribution over the quadrants was not equal (*p* < 0.001). Post hoc analysis with a Bonferroni adjustment revealed differences between AM and PL (*p* < 0.001) and between AM and AL (*p* = 0.005)

## Discussion

The aim of this study was to assess the accuracy of two different measuring methods for cement penetration and cement distribution of the tibia using CT scan images in patients with a total knee arthroplasty.

### Intra-rater variability and inter-rater variability

The variability of the manual method was very low. Bland Altman plots showed that the variability of the manual method can be explained by the lack of agreement at 3 mm depth underneath the tibial tray. Cement is more scattered at that depth and therefore difficult to obtain visually. However, the ICC at 1 and 5 mm depth was also low. Which was in contrast to the variability of the matlab method. Results of the intra-rater variability and inter-rater variability were highly superior over the manual method at all depths. Portney and Watkins [17] suggested that ICC values of > 0.90 represent an excellent reliability. Therefore, we think that, with this user-friendly matlab approach, we present a reliable method for assessing the cement mantle, which can be used for clinical evaluation and for scientific studies.

### Cement penetration

Walker et al.(1984) already stated that optimal fixation requires penetration of cement into the proximal tibia of 3 to 4 mm. In recent years a good penetration of cement into the trabecular bone is still a requirement for a solid fixation strength of the prosthesis [12, 15].

In this study we used the common surface cementing technique for the tibial baseplate. With this method the cement penetration at a depth of 1 mm was very good. In contrast, at 3 mm depth measured with the matlab method a median of 39% in the antero-medial quarter to 53% in the antero-lateral quarter of the trabecular bony surface was penetrated with cement. At 5 mm depth only 15% of the antero-medial and 25% of the postero-lateral quarter was filled with cement. Further research is necessary to optimize the cementing technique for a better fixation of the tibia baseplate with the trabecular bone. If these cementing techniques are evaluated to examine the cement penetration, then this CT scan method provides a reliable measurement method for penetration in vivo.

### Cement distribution

The volume of cement penetrating into the bone is less predictive of failure than the mean penetration, which indicates the importance of a good distribution of the cement [13].

At 1 mm beneath the tibial tray, all four quadrants had an equal distribution of cement over an equal percentage of the trabecular bone surface.

At 3 and 5 mm depth, there was significantly less cement penetration in the anterior-medial quadrant. Perhaps this is due to the holes of the pins that were used for fixation of the tibial cutting guide and for the tibial sizing plate. Both instruments were fixated temporarily on the anterior-medial side, and these holes frequently penetrates the cortex of the proximal tibia. It seems possible that by compressing the cement, an amount of the cement is pressed through these holes out of the tibia. More research is needed to support the explanation for these divergent results.

### Limitations

A limitation of our study is that with this CT technique, it is impossible to differentiate between bulk cement, such as filled bony defects or cysts, and trabecular bone that is interlocked with cement. When cement is pressurized into trabecular bone, the cement fills up the space between the trabeculae, which enlarges the trabecular bone’s density and improves its homogeneity. As a result, the homogeneity value of cement-penetrated trabecular bone is almost the same as the homogeneity value of pure cement. With this result, we can assume that the density of cement-penetrated trabecular bone equals the density of pure cement. To differentiate between them, we would have to use micro-CT, which cannot be used in vivo. We therefore accepted this limitation, since this study aimed to develop and validate a measuring technique that can be used in clinical practice.

A second limitation is that it is not possible to assess the space just surrounding the stem of the prosthesis due to the scattering of the metal. This even was the case with the prosthesis that we used in this study, which has a tibial component of titanium alloy and allowed post-surgery CT scanning of the cement mantle without excessive metal scattering effects of the prosthesis [13].

However, the scattering had the same influence on the data at the three different levels, and hence it did not influence the results of this study.

## Conclusions

Distribution and penetration of cement in the proximal tibia in a total knee arthroplasty can be measured reliably with CT in combination with the matlab method presented in this manuscript. This method can be used for clinical purposes as well as for scientific research.

## Supplementary information

**Additional file 1.**

## Data Availability

The datasets used and/or analysed during the current study are available from the corresponding author on reasonable request.

## Abbreviations

A-L: Antero-lateral
A-M: Antero-medial
BMI: Body mass index
CT: Computer tomography
HU: Hounsfield Unit
ICC: Intraclass correlation coefficient
IQR: Interquartile range
kV: Kilovolt
mA: Milliampere
mm: Millimeter
PACS: Picture archiving and communication system
P-L: Postero-lateral
P-M: Postero-medial
PMMA: Polymethylmethacrylaat
SD: Standard deviation
SPSS: Statistical package for the social sciences
TKA: Total knee arthroplasty
