# Supplementary material for: Reliability of two different measuring techniques with computer tomography for penetration and distribution of cement in the proximal tibia after total knee arthroplasty
Source: BMC Musculoskelet Disord. 2020 Jun 12;21:374. doi: 10.1186/s12891-020-03390-3 (PMC7291566; doi:10.1186/s12891-020-03390-3)
Supplement: Supplementary file 1 — Additional file 1. [file 12891_2020_3390_MOESM1_ESM.zip › CalculateCementR4.pdf]

```matlab
%% Initialize
close all
clearvars -except XLfile
imtool close all
%% check and open Excel file
if exist ('XLfile', 'var') ==0
    FileOpener
else
    allRes=readmatrix(XLfile);
end
%% Determine baseplate
postcalibrationBaseplate
%% Determine cement and quadrants
postcalibration3DIMtool
satis=0;
while satis==0
    close all
    tool = imtool3D(drieDcement)
    pos=get(gcf,'Position');
    pos(2)=pos(2)-0.2;
    set(gcf,'Position',pos)
    selectROI


    happy = questdlg('Are you satisfied with these quadrants?',...
        'Check',...
        'Yes','No','Yes');

    switch happy
        case 'No'
            satis = 0;
        case 'Yes'
            satis = 1;
    end

end
%% Geef zijde aan
side = questdlg('Of which side is this scan?',...
    'Choose side',...
    'Left','Right','Right');
switch side
    case 'Left'
        zijde = 0;
    case 'Right'
        zijde = 1;
end
%% Find cement per quadrant
%Find start horizontal line
for i=1:length(forUse)
    if isempty(find(horMask(:,i)==1))==0
        startHor=i;
        break
    end
end
cemArea=[];
totArea=[];
exCemArea=[];
for l=1:3
    left=1; %1=true 2=false
    top=1;  %1=true 2=false
    cem=zeros(2,2);
    tot=zeros(2,2);
```

```
    exCem=zeros(2,2);
    for i=1:length(forUse)      %% Loop over rows of pixels
        left=1;
        if i<find(horMask(:,startHor)) %%If row starts under or above horizontal line
            top=1;
        else
            top=2;
        end

        for j=1:length(forUse) %% Loop per pixel
            vox=forUse(i,j,l);
            if vox==1
                tot(top,left)=tot(top,left)+1;      %% add pixel to total
            end
            if vox==2
                tot(top,left)=tot(top,left)+1;      %% add pixel to total
                cem(top,left)=cem(top,left)+1;      %% add pixel to cement total
            end
            if vox==3
                exCem(top,left)=exCem(top,left)+1;  %%add pixel to excess cement
            end
            if verMask(i,j)==1 && left==1       %% change quadrant from left to right
                left=2;
            end
            if horMask(i,j)==1                  %% change quadrant from top to bottom
or bottom to top
                if top==1
                    top=2;
                else
                    top=1;
                end
            end
        end
    end
    cemArea(:,:,1)=cem;
    totArea(:,:,1)=tot;
    exCemArea(:,:,1)=exCem;
    %%  Quadranten wegschrijven
    if zijde==1
        AM.Cement=cemArea(1,1,:);
        AM.Total=totArea(1,1,:);
        AM.exCem=exCemArea(1,1,:);

        AL.Cement=cemArea(1,2,:);
        AL.Total=totArea(1,2,:);
        AL.exCem=exCemArea(1,2,:);

        PM.Cement=cemArea(2,1,:);
        PM.Total=totArea(2,1,:);
        PM.exCem=exCemArea(2,1,:);

        PL.Cement=cemArea(2,2,:);
        PL.Total=totArea(2,2,:);
        PL.exCem=exCemArea(2,2,:);
    else
        AM.Cement=cemArea(1,2,:);
        AM.Total=totArea(1,2,:);
        AM.exCem=exCemArea(1,2,:);

        AL.Cement=cemArea(1,1,:);
        AL.Total=totArea(1,1,:);
        AL.exCem=exCemArea(1,1,:);
```

```
        PM.Cement=cemArea(2,2,:);
        PM.Total=totArea(2,2,:);
        PM.exCem=exCemArea(2,2,:);

        PL.Cement=cemArea(2,1,:);
        PL.Total=totArea(2,1,:);
        PL.exCem=exCemArea(1,2,:);
    end
%% Percentages berekenen
if ~isempty(allRes)
    ind=find(allRes(:,1)==patNum);
else
    ind=[];
end
Percentage.AM = (AM.Cement./AM.Total)*100;
Percentage.AL = (AL.Cement./AL.Total)*100;
Percentage.PM = (PM.Cement./PM.Total)*100;
Percentage.PL = (PL.Cement./PL.Total)*100;
Percentage.exAM=(AM.exCem./(AM.exCem+AM.Cement))*100;
Percentage.exAL=(AL.exCem./(AL.exCem+AL.Cement))*100;
Percentage.exPM=(PM.exCem./(PM.exCem+PM.Cement))*100;
Percentage.exPL=(PL.exCem./(PL.exCem+PL.Cement))*100;
if isempty(ind)
    allRes=[allRes;nan(1,size(allRes,2))];
    allRes(end,[1 l+1 l+4 l+7 l+10 l+13 l+16 l+19 l+22])=[patNum Percentage.AM
Percentage.AL Percentage.PM Percentage.PL Percentage.exAM Percentage.exAL
Percentage.exPM Percentage.exPL];
else
    allRes(ind,[1 l+1 l+4 l+7 l+10 l+13 l+16 l+19 l+22])=[patNum Percentage.AM
Percentage.AL Percentage.PM Percentage.PL Percentage.exAM Percentage.exAL
Percentage.exPM Percentage.exPL];
end
 sortrows(allRes);
end
%% Plot
figure
plot(allRes(ind,2:4),'r*-')
hold on
plot(allRes(ind,5:7),'b*-')
hold on
plot(allRes(ind,8:10),'g*-')
hold on
plot(allRes(ind,11:13),'k*-')
ylim([0 100])
legend('Percentage cementpenetratie AM','Percentage cementpenetratie AL','Percentage
cementpenetratie PM','Percentage cementpenetratie PL')
%% Write to excel
varNames={'patNum' ;'AM_1' ;'AM_2'; 'AM_3' ;'AL_1' ;'AL_2'; 'AL_3' ;'PM_1'; 'PM_2'
;'PM_3' ;'PL_1' ;'PL_2'
;'PL_3';'exAM_1';'exAM_2';'exAM_3';'exAL_1';'exAL_2';'exAL_3';'exPM_1';'exPM_2';'exPM_
3';'exPL_1';'exPL_2';'exPL_3'};
table=array2table(allRes,'VariableNames',varNames);
writetable(table,XLfile);
```
