# Supplementary material for: Reliability of two different measuring techniques with computer tomography for penetration and distribution of cement in the proximal tibia after total knee arthroplasty
Source: BMC Musculoskelet Disord. 2020 Jun 12;21:374. doi: 10.1186/s12891-020-03390-3 (PMC7291566; doi:10.1186/s12891-020-03390-3)
Supplement: Supplementary file 1 — Additional file 1. [file 12891_2020_3390_MOESM1_ESM.zip › FileOpenerR4.pdf]

```matlab
%% check and open Excel file
newFile=askNew();
if newFile
    XLfile=askFilename();
    allRes=[];
    writetable(array2table([]),XLfile);
else
    XLfile=askDir()
    checkOpen();
    allRes=readmatrix(XLfile);
end
function newFile = askNew()
new = questdlg('Would you like to use a new excel file or an existing one?',...
    'File for data',...
    'New','Existing','Existing');
switch new
    case 'New'
        newFile = 1;
    case 'Existing'
        newFile = 0;
end
end
function checkOpen()
try
    %Check if an Excel server is running
    ex = actxGetRunningServer('Excel.Application');
catch ME
    disp(ME.message)
end
if exist('ex','var')
    %Get the names of all open Excel files
    wbs = ex.Workbooks;
    %List the entire path of all excel workbooks that are currently open
    for i = 1:wbs.Count
        openFiles(i,:)=wbs.Item(i).FullName;
        if contains(openFiles,XLfile)
            uiwait(msgbox('Close excel file','!!! INFORMATION !!!','help'));
        end
    end
end
end
function filename=askFilename()
    filename= inputdlg('Enter desired file name (include extension .xlsx)','Input file
name',[1 50]);
    path= uigetdir('','Choose directory');
    filename=[path '\' filename{1}];
end
function filename=askDir()
    [filename,path]= uigetfile('.xlsx','Select excel data');
    filename=[path,filename];
end
```
