# Supplementary material for: Reliability of two different measuring techniques with computer tomography for penetration and distribution of cement in the proximal tibia after total knee arthroplasty
Source: BMC Musculoskelet Disord. 2020 Jun 12;21:374. doi: 10.1186/s12891-020-03390-3 (PMC7291566; doi:10.1186/s12891-020-03390-3)
Supplement: Supplementary file 1 — Additional file 1. [file 12891_2020_3390_MOESM1_ESM.zip › imtool3DR4.pdf]

```
classdef imtool3D < handle
    %This is a image slice viewer with built in scroll, contrast, zoom and
    %ROI tools.
    %
    %   Use this class to place a self-contained image viewing panel within
    %   a GUI (or any figure). Similar to imtool but with slice scrolling.
    %   Only designed to view grayscale (intensity) images. Use the mouse
    %   to control how the image is displayed. A left click allows window
    %   and leveling, a right click is for panning, and a middle click is
    %   for zooming. Also the scroll wheel can be used to scroll through
    %   slices.
    %----------------------------------------------------------------------
    %Inputs:
    %
    %   I           An m x n x k image array of grayscale values. Default
    %               is a 100x100x3 random noise image.
    %   position    The position of the panel containing the image and all
    %               the tools. Format is [xmin ymin width height]. Default
    %               position is [0 0 1 1] (units = normalized). See the
    %               setPostion and setUnits methods to change the postion
    %               or units.
    %   h           Handle of the parent figure. If no handles is provided,
    %               a new figure will be created.
    %   range       The display range of the image. Format is [min max].
    %               The range can be adjusted with the contrast tool or
    %               with the setRange method. Default is [min(I) max(I)].
    %----------------------------------------------------------------------
    %Output:
    %
    %   tool        The imtool3D object. Use this object as input to the
    %               class methods described below.
    %----------------------------------------------------------------------
    %Constructor Syntax
    %
    %tool = imtool3d() creates an imtool3D panel in the current figure with
    %a random noise image. Returns the imtool3D object.
    %
    %tool = imtool3d(I) sets the image of the imtool3D panel.
    %
    %tool = imtool3D(I,position) sets the position of the imtool3D panel
    %within the current figure. The default units are normalized.
    %
    %tool = imtool3D(I,position,h) puts the imtool3D panel in the figure
    %specified by the handle h.
    %
    %tool = imtool3D(I,position,h,range) sets the display range of the
    %image according to range=[min max].
    %
    %tool = imtool3D(I,position,h,range,tools) lets the scroll wheel
    %properly sync if you are displaying multiple imtool3D objects in the
    %same figure.
    %
    %Note that you can pass an empty matrix for any input variable to have
    %the constructor use default values. ex. tool=imtool3D([],[],h,[]).
    %----------------------------------------------------------------------
    %Methods:
    %
    %   setImage(tool, I) displays a new image.
    %
    %   I = getimage(tool) returns the image being shown by the tool
    %
    %   setPostion(tool,position) sets the position of tool.
```

```
%
%   position = getPosition(tool) returns the position of the tool
%   relative to its parent figure.
%
%   setUnits(tool,Units) sets the units of the position of tool. See
%   uipanel properties for possible unit strings.
%
%   units = getUnits(tool) returns the units of used for the position
%   of the tool.
%
%   handles = getHandles(tool) returns a structured variable, handles,
%   which contains all the handles to the various objects used by
%   imtool3D.
%
%   setDisplayRange(tool,range) sets the display range of the image.
%   see the 'Clim' property of an Axes object for details.
%
%   range=getDisplayRange(tool) returns the current display range of
%   the image.
%
%   setWindowLevel(tool,W,L) sets the display range of the image in
%   terms of its window (diff(range)) and level (mean(range)).
%
%   [W,L] = getWindowLevel(tool) returns the display range of the image
%   in terms of its window (W) and level (L)
%
%   ROI = getCurrentROI(tool) returns info about the currently selected
%   region of interest (ROI). If no ROI is currently selected, the
%   method returns an empty matrix. ROI is a structured variable with
%   the following fields:
%       -ROI.mask is a binary mask that defines the pixels within the
%       ROI.
%       -ROI.stats is a structured variable containing stats about the
%       ROI. Included stats are, Area, Perimeter, MaxIntensity,
%       MinIntensity, MeanIntensity, and STD.
%
%   setCurrentSlice(tool,slice) sets the current displayed slice.
%
%   slice = getCurrentSlice(tool) returns the currently displayed
%   slice.
%
%----------------------------------------------------------------------
%Notes:
%
%   Author: Justin Solomon, July, 26 2013
%
%   Contact: justin.solomon@duke.edu
%
%   Current Version 2.1
%   Version Notes:
%                   1.1-added method to get information about the
%                   currently selected ROI.
%
%                   2.0- Completely redesigned the tool. Window and
%                   leveleing, pan, and zoom are now done with the
%                   mouse as is standard in most medical image viewers.
%                   Also the overall astestic design of the tool is
%                   improved with a new black theme. Added ability to
%                   change the colormap of the image. Also when
%                   resizing the figure, the tool behaves better and
%                   maintains maximum viewing area for the image while
%                   keeping the tool buttons correctly sized.
```

```
    %                   IMPORTANT: Any code that worked with the version
    %                   1.0 may not be compatible with version 2.0.
    %
    %                   2.1- Added crop tool, help button, and button that
    %                   resets the pan and zoom settings to show the entire
    %                   image (useful when you're zoomed in and you just
    %                   want to zoom out quickly. Also made the window and
    %                   level adjustable by draging the lines on the
    %                   histogram
    %
    %   Created in MATLAB_R2013b
    %
    %   Requires the image processing toolbox

    properties (SetAccess = private, GetAccess = private)
        I            %Image data (MxNxK) matrix of image data
        handles      %Structured variable with all the handles
        centers      %list of bin centers for histogram
        ROIhandles   %list of ROI handles
        CurrentROI   %Currently selected ROI
    end

    methods

        function tool = imtool3D(varargin)  %Constructor

            %Check the inputs and set things appropriately
            switch nargin
                case 0  %tool = imtool3d()
                    I=random('unif',-50,50,[100 100 3]);
                    position=[0 0 1 1]; h=figure;
set(h,'Toolbar','none','Menubar','none')
                    range=[-50 50]; tools=[];
                case 1  %tool = imtool3d(I)
                    I=varargin{1}; position=[0 0 1 1]; h=figure;
set(h,'Toolbar','none','Menubar','none')
                    range=[min(I(:)) max(I(:))]; tools=[];
                case 2  %tool = imtool3d(I,position)
                    I=varargin{1}; position=varargin{2}; h=figure;
set(h,'Toolbar','none','Menubar','none')
                    range=[min(I(:)) max(I(:))]; tools=[];
                case 3  %tool = imtool3d(I,position,h)
                    I=varargin{1}; position=varargin{2}; h=varargin{3};
                    range=[min(I(:)) max(I(:))]; tools=[];
                case 4  %tool = imtool3d(I,position,h,range)
                    I=varargin{1}; position=varargin{2}; h=varargin{3};
                    range=varargin{4}; tools=[];
                case 5  %tool = imtool3d(I,position,h,range,tools)
                    I=varargin{1}; position=varargin{2}; h=varargin{3};
                    range=varargin{4}; tools=varargin{5};
            end

            if isempty(I)
                I=random('unif',-50,50,[100 100 3]);
            end

            if isempty(position)
                position=[0 0 1 1];
            end

            if isempty(h)
                h=figure;
```

```
            end

            if isempty(range)
                range=[min(I(:)) max(I(:))];
            end

            %Make the aspect ratio of the figure match that of the image
            if nargin<3
                set(h,'Units','Pixels');
                pos=get(h,'Position');
                Af=pos(3)/pos(4);   %Aspect Ratio of the figure
                AI=size(I,2)/size(I,1); %Aspect Ratio of the image
                if Af>AI    %Figure is too wide, make it taller to match
                   pos(4)=pos(3)/AI;
                elseif Af<AI    %Figure is too long, make it wider to match
                    pos(3)=AI*pos(4);
                end
                set(h,'Position',pos)
                set(h,'Units','normalized');
            end


            I=double(I);

            %-------------------------------------------------------------
            tool.I          = I;
            tool.handles.fig=h;
            ROIhandles=[];
            CurrentROI=[];

            %Create the panels and slider
            w=30; %Pixel width of the side panels
            h=110; %Pixel height of the histogram panel
            wbutt=20; %Pixel size of the buttons
            tool.handles.Panels.Large   =
uipanel(tool.handles.fig,'Position',position,'Title','','Tag','imtool3D');
set(tool.handles.Panels.Large,'Units','Pixels');
pos=get(tool.handles.Panels.Large,'Position');
set(tool.handles.Panels.Large,'Units','normalized');
            tool.handles.Panels.Hist   =
uipanel(tool.handles.Panels.Large,'Units','Pixels','Position',[w pos(4)-w-h pos(3)-2*w
h],'Title','');
            tool.handles.Panels.Image   =
uipanel(tool.handles.Panels.Large,'Units','Pixels','Position',[w w pos(3)-2*w pos(4)-
2*w],'Title','');
            tool.handles.Panels.Tools   =
uipanel(tool.handles.Panels.Large,'Units','Pixels','Position',[0 pos(4)-w pos(3)
w],'Title','');
            tool.handles.Panels.ROItools    =
uipanel(tool.handles.Panels.Large,'Units','Pixels','Position',[pos(3)-w  w w pos(4)-
2*w],'Title','');
            tool.handles.Panels.Slider  =
uipanel(tool.handles.Panels.Large,'Units','Pixels','Position',[0 w w pos(4)-
2*w],'Title','');
            tool.handles.Panels.Info   =
uipanel(tool.handles.Panels.Large,'Units','Pixels','Position',[0 0 pos(3)
w],'Title','');
            try

set(cell2mat(struct2cell(tool.handles.Panels)),'BackgroundColor','k','ForegroundColor'
,'w','HighlightColor','k')
            catch
```

```
                  objarr=struct2cell(tool.handles.Panels);
                  objarr=[objarr{:}];
set(objarr,'BackgroundColor','k','ForegroundColor','w','HighlightColor','k');
            end


            %Create Slider for scrolling through image stack
            tool.handles.Slider         =
uicontrol(tool.handles.Panels.Slider,'Style','Slider','Units','Normalized','Position',
[0 0 1 1],'TooltipString','Change Slice (can use scroll wheel also)');
            setupSlider(tool)
            fun=@(scr,evnt)multipleScrollWheel(scr,evnt,[tool tools]);
            %fun=@(scr,evnt) scrollWheel(scr,evnt,tool);
%             fncs=get(tool.handles.fig,'WindowScrollWheelFcn');
%             if isempty(fncs)
%                 fncs{end+1}=fun;
%             elseif ~iscell(fncs)
%                 fncs={fncs};
%                 fncs{end+1}=fun;
%             else
%                 fncs{end+1}=fun;
%             end
            set(tool.handles.fig,'WindowScrollWheelFcn',fun);


            %Create image axis
            tool.handles.Axes           =   axes('Position',[0 0 1
1],'Parent',tool.handles.Panels.Image,'Color','none');
            tool.handles.I              =   imshow(I(:,:,1),range);
            set(tool.handles.Axes,'Position',[0 0 1
1],'Color','none','XColor','r','YColor','r','GridLineStyle','--
','LineWidth',1.5,'XTickLabel','','YTickLabel','');
            axis off
            grid off
            axis fill

            %Set up image info display

tool.handles.Info=uicontrol(tool.handles.Panels.Info,'Style','text','String','(x,y)
val','Units','Normalized','Position',[0 .1 .5
.8],'BackgroundColor','k','ForegroundColor','w','FontSize',12,'HorizontalAlignment','L
eft');

tool.handles.ROIinfo=uicontrol(tool.handles.Panels.Info,'Style','text','String','STD:
Mean:                    ','Units','Normalized','Position',[.5 .1 .5
.8],'BackgroundColor','k','ForegroundColor','w','FontSize',12,'HorizontalAlignment','R
ight');
            fun=@(src,evnt)getImageInfo(src,evnt,tool);
            set(tool.handles.fig,'WindowButtonMotionFcn',fun);

tool.handles.SliceText=uicontrol(tool.handles.Panels.Tools,'Style','text','String',['1
/' num2str(size(I,3))],'Units','Normalized','Position',[.5 .1 .48
.8],'BackgroundColor','k','ForegroundColor','w','FontSize',12,'HorizontalAlignment','R
ight');


            %Set up mouse button controls
            fun=@(hObject,eventdata) imageButtonDownFunction(hObject,eventdata,tool);
            set(tool.handles.I,'ButtonDownFcn',fun)

            %create the tool buttons
```

```
            wp=w;
            w=wbutt;
            buff=(wp-w)/2;

            %Create the histogram plot
            tool.handles.HistAxes           =   axes('Position',[.025 .15 .95
.55],'Parent',tool.handles.Panels.Hist);
            im=tool.I(:,:,1);
            centers=linspace(min(I(:)),max(I(:)),256);
            nelements=hist(im(:),centers); nelements=nelements./max(nelements);
            tool.handles.HistLine=plot(centers,nelements,'-w','LineWidth',1);

set(tool.handles.HistAxes,'Color','none','XColor','w','YColor','w','FontSize',9,'YTick
',[])
            axis on
            hold on
            axis fill
            xlim(get(gca,'Xlim'))
            tool.handles.Histrange(1)=plot([range(1) range(1) range(1)],[0 .5 1],'.-
r');
            tool.handles.Histrange(2)=plot([range(2) range(2) range(2)],[0 .5 1],'.-
r');
            tool.handles.Histrange(3)=plot([mean(range) mean(range) mean(range)],[0 .5
1],'.--r');
            tool.handles.HistImageAxes           =   axes('Position',[.025 .75 .95
.2],'Parent',tool.handles.Panels.Hist);
            set(tool.handles.HistImageAxes,'Units','Pixels');
pos=get(tool.handles.HistImageAxes,'Position');
set(tool.handles.HistImageAxes,'Units','Normalized');
            tool.handles.HistImage=imshow(repmat(centers,[round(pos(4)) 1]),range);

set(tool.handles.HistImageAxes,'XColor','w','YColor','w','XTick',[],'YTick',[])
            axis on;
            box on;
            axis normal
            tool.centers=centers;
            fun = @(hObject,evnt)histogramButtonDownFunction(hObject,evnt,tool,1);
            set(tool.handles.Histrange(1),'ButtonDownFcn',fun);
            fun = @(hObject,evnt)histogramButtonDownFunction(hObject,evnt,tool,2);
            set(tool.handles.Histrange(2),'ButtonDownFcn',fun);
            fun = @(hObject,evnt)histogramButtonDownFunction(hObject,evnt,tool,3);
            set(tool.handles.Histrange(3),'ButtonDownFcn',fun);

            %Create histogram checkbox
            tool.handles.Tools.Hist     =
uicontrol(tool.handles.Panels.Tools,'Style','Checkbox','String','Hist?','Position',[bu
ff buff 2.5*w w],'TooltipString','Show
Histogram','BackgroundColor','k','ForegroundColor','w');
            fun=@(hObject,evnt) ShowHistogram(hObject,evnt,tool,wp,h);
            set(tool.handles.Tools.Hist,'Callback',fun)
            lp=buff+2.5*w;


            %Set up the resize function
            fun=@(x,y) panelResizeFunction(x,y,tool,wp,h,wbutt);
            set(tool.handles.Panels.Large,'ResizeFcn',fun)

            %Create window and level boxes
            tool.handles.Tools.TW       =
uicontrol(tool.handles.Panels.Tools,'Style','text','String','W','Position',[lp+buff
buff w w],'BackgroundColor','k','ForegroundColor','w','TooltipString','Window Width');
```

```
            tool.handles.Tools.W        =
uicontrol(tool.handles.Panels.Tools,'Style','Edit','String',num2str(range(2)-
range(1)),'Position',[lp+buff+w buff 2*w w],'TooltipString','Window Width');
            tool.handles.Tools.TL       =
uicontrol(tool.handles.Panels.Tools,'Style','text','String','L','Position',[lp+2*buff+
3*w buff w w],'BackgroundColor','k','ForegroundColor','w','TooltipString','Window
Level');
            tool.handles.Tools.L        =
uicontrol(tool.handles.Panels.Tools,'Style','Edit','String',num2str(mean(range)),'Posi
tion',[lp+2*buff+4*w buff 2*w w],'TooltipString','Window Level');
            lp=lp+buff+7*w;

            %Creat window and level callbacks
            fun=@(hobject,evnt) WindowLevel_callback(hobject,evnt,tool);
            set(tool.handles.Tools.W,'Callback',fun);
            set(tool.handles.Tools.L,'Callback',fun);

            %Create view restore button
            tool.handles.Tools.ViewRestore           =
uicontrol(tool.handles.Panels.Tools,'Style','pushbutton','String','','Position',[lp
buff w w],'TooltipString','Reset Pan and Zoom');
            [iptdir, MATLABdir] = ipticondir;
            icon_save = makeToolbarIconFromPNG([iptdir '/overview_zoom_in.png']);
            set(tool.handles.Tools.ViewRestore,'CData',icon_save);
            fun=@(hobject,evnt) resetViewCallback(hobject,evnt,tool);
            set(tool.handles.Tools.ViewRestore,'Callback',fun)
            lp=lp+w+2*buff;

            %Create grid checkbox and grid lines
            axes(tool.handles.Axes)
            tool.handles.Tools.Grid           =
uicontrol(tool.handles.Panels.Tools,'Style','checkbox','String','Grid?','Position',[lp
buff 2.5*w w],'BackgroundColor','k','ForegroundColor','w');
            nGrid=7;
            nMinor=4;
            x=linspace(1,size(I,2),nGrid);
            y=linspace(1,size(I,1),nGrid);
            hold on;
            tool.handles.grid=[];
            gColor=[255 38 38]./256;
            mColor=[255 102 102]./256;
            for i=1:nGrid
                tool.handles.grid(end+1)=plot([.5 size(I,2)-.5],[y(i) y(i)],'-
','LineWidth',1.2,'HitTest','off','Color',gColor);
                tool.handles.grid(end+1)=plot([x(i) x(i)],[.5 size(I,1)-.5],'-
','LineWidth',1.2,'HitTest','off','Color',gColor);
                if i<nGrid
                    xm=linspace(x(i),x(i+1),nMinor+2); xm=xm(2:end-1);
                    ym=linspace(y(i),y(i+1),nMinor+2); ym=ym(2:end-1);
                    for j=1:nMinor
                        tool.handles.grid(end+1)=plot([.5 size(I,2)-.5],[ym(j)
ym(j)],'-r','LineWidth',.9,'HitTest','off','Color',mColor);
                        tool.handles.grid(end+1)=plot([xm(j) xm(j)],[.5 size(I,1)-
.5],'-r','LineWidth',.9,'HitTest','off','Color',mColor);
                    end
                end
            end

tool.handles.grid(end+1)=scatter(.5+size(I,2)/2,.5+size(I,1)/2,'r','filled');
            set(tool.handles.grid,'Visible','off')
            fun=@(hObject,evnt) toggleGrid(hObject,evnt,tool);
            set(tool.handles.Tools.Grid,'Callback',fun)
```

```
            set(tool.handles.Tools.Grid,'TooltipString','Toggle Gridlines')
            lp=lp+2.5*w;

            %Create colormap pulldown menu

mapNames={'Gray','Hot','Jet','HSV','Cool','Spring','Summer','Autumn','Winter','Bone','
Copper','Pink','Lines','colorcube','flag','prism','white'};
            tool.handles.Tools.Color          =
uicontrol(tool.handles.Panels.Tools,'Style','popupmenu','String',mapNames,'Position',[
lp buff 4*w w]);
            fun=@(hObject,evnt) changeColormap(hObject,evnt,tool);
            set(tool.handles.Tools.Color,'Callback',fun)
            set(tool.handles.Tools.Color,'TooltipString','Select a colormap')
            lp=lp+4*w;

            %Create save button
            tool.handles.Tools.Save           =
uicontrol(tool.handles.Panels.Tools,'Style','pushbutton','String','','Position',[lp
buff w w]);
            icon_save = makeToolbarIconFromPNG([MATLABdir '/file_save.png']);
            set(tool.handles.Tools.Save,'CData',icon_save);
            lp=lp+w;
            tool.handles.Tools.SaveOptions    =
uicontrol(tool.handles.Panels.Tools,'Style','popupmenu','String',{'as slice','as
stack'},'Position',[lp buff 5*w w]);
            fun=@(hObject,evnt) saveImage(hObject,evnt,tool);
            set(tool.handles.Tools.Save,'Callback',fun)
            set(tool.handles.Tools.Save,'TooltipString','Save image as slice or tiff
stack')


            %Create Circle ROI button
            tool.handles.Tools.CircleROI           =
uicontrol(tool.handles.Panels.ROItools,'Style','pushbutton','String','','Position',[bu
ff buff w w],'TooltipString','Create Elliptical ROI');
            icon_ellipse = makeToolbarIconFromPNG([MATLABdir
'/tool_shape_ellipse.png']);
            set(tool.handles.Tools.CircleROI,'Cdata',icon_ellipse)
            fun=@(hObject,evnt) measureImageCallback(hObject,evnt,tool,'ellipse');
            set(tool.handles.Tools.CircleROI,'Callback',fun)

            %Create Square ROI button
            tool.handles.Tools.SquareROI           =
uicontrol(tool.handles.Panels.ROItools,'Style','pushbutton','String','','Position',[bu
ff buff+w w w],'TooltipString','Create Rectangular ROI');
            icon_rect = makeToolbarIconFromPNG([MATLABdir
'/tool_shape_rectangle.png']);
            set(tool.handles.Tools.SquareROI,'Cdata',icon_rect)
            fun=@(hObject,evnt) measureImageCallback(hObject,evnt,tool,'rectangle');
            set(tool.handles.Tools.SquareROI,'Callback',fun)

            %Create Polygon ROI button
            tool.handles.Tools.PolyROI             =
uicontrol(tool.handles.Panels.ROItools,'Style','pushbutton','String','\_/','Position',
[buff buff+2*w w w],'TooltipString','Create Polygon ROI');
            fun=@(hObject,evnt) measureImageCallback(hObject,evnt,tool,'polygon');
            set(tool.handles.Tools.PolyROI,'Callback',fun)

            %Create Delete Button
            tool.handles.Tools.DeleteROI          =
uicontrol(tool.handles.Panels.ROItools,'Style','pushbutton','String','X','Position',[b
uff buff+3*w w w],'TooltipString','Delete ROI','ForegroundColor','r');
```

```
            fun=@(hObject,evnt) deleteCurrentROI(hObject,evnt,tool);
            set(tool.handles.Tools.DeleteROI,'Callback',fun)

            %Create Export ROI Button
            tool.handles.Tools.ExportROI           =
uicontrol(tool.handles.Panels.ROItools,'Style','pushbutton','String','-
>','Position',[buff buff+4*w w w],'TooltipString','Export ROI to
Workspace','ForegroundColor','k');
            fun=@(hObject,evnt) exportROI(hObject,evnt,tool);
            set(tool.handles.Tools.ExportROI,'Callback',fun)

            %Create Ruler button
            tool.handles.Tools.Ruler             =
uicontrol(tool.handles.Panels.ROItools,'Style','pushbutton','String','','Position',[bu
ff buff+6*w w w],'TooltipString','Measure Distance');
            icon_distance = makeToolbarIconFromPNG([MATLABdir '/tool_line.png']);
            set(tool.handles.Tools.Ruler,'CData',icon_distance);
            fun=@(hObject,evnt) measureImageCallback(hObject,evnt,tool,'ruler');
            set(tool.handles.Tools.Ruler,'Callback',fun)

            %Create Line Profile button
            tool.handles.Tools.Profile             =
uicontrol(tool.handles.Panels.ROItools,'Style','pushbutton','String','','Position',[bu
ff buff+7*w w w],'TooltipString','Get Line Profile');
            icon_profile = makeToolbarIconFromPNG([iptdir '/profile.png']);
            set(tool.handles.Tools.Profile,'Cdata',icon_profile)
            fun=@(hObject,evnt) measureImageCallback(hObject,evnt,tool,'profile');
            set(tool.handles.Tools.Profile,'Callback',fun)

            %Create Crop tool button
            tool.handles.Tools.Crop             =
uicontrol(tool.handles.Panels.ROItools,'Style','pushbutton','String','','Position',[bu
ff buff+9*w w w],'TooltipString','Crop Image');
            icon_profile = makeToolbarIconFromPNG([iptdir '/crop_tool.png']);
            set(tool.handles.Tools.Crop ,'Cdata',icon_profile)
            fun=@(hObject,evnt) CropImageCallback(hObject,evnt,tool);
            set(tool.handles.Tools.Crop ,'Callback',fun)


            %Create Help Button
            pos=get(tool.handles.Panels.ROItools,'Position');
            tool.handles.Tools.Help             =
uicontrol(tool.handles.Panels.ROItools,'Style','pushbutton','String','?','Position',[b
uff pos(4)-w-buff w w],'TooltipString','Help with imtool3D');
            fun=@(hObject,evnt) displayHelp(hObject,evnt,tool);
            set(tool.handles.Tools.Help,'Callback',fun)

            %Set font size of all the tool objects
            try

set(cell2mat(struct2cell(tool.handles.Tools)),'FontSize',9,'Units','Pixels')
            catch
                objarr=struct2cell(tool.handles.Tools);
                objarr=[objarr{:}];
                set(objarr,'FontSize',9,'Units','Pixels')
            end

        end

        function setPosition(tool,position)
            set(tool.handles.Panels.Large,'Position',position)
        end
```

```
function position = getPosition(tool)
    position = get(tool.handles.Panels.Large,'Position');
end

function setUnits(tool,units)
    set(tool.handles.Panels.Large,'Units',units)
end

function units = getUnits(tool)
    units = get(tool.handles.Panels.Large,'Units')
end

function setImage(varargin)
    switch nargin
        case 1
            tool=varargin{1}; I=random('unif',-50,50,[100 100 3]);
            range=[-50 50];
        case 2
            tool=varargin{1}; I=varargin{2};
            range=[min(I(:)) max(I(:))];
        case 3
            tool=varargin{1}; I=varargin{2};
            range=varargin{3};
    end

    if isempty(I)
        I=random('unif',-50,50,[100 100 3]);
    end
    if isempty(range)
        range=[min(I(:)) max(I(:))];
    end

    tool.I=I;

    %Update the histogram
    im=tool.I(:,:,1);
    tool.centers=linspace(min(I(:)),max(I(:)),256);
    nelements=hist(im(:),tool.centers); nelements=nelements./max(nelements);
    set(tool.handles.HistLine,'XData',tool.centers,'YData',nelements);
    axes(tool.handles.HistAxes);
    xlim([tool.centers(1) tool.centers(end)])
    axis fill

    %Update the window and level
    setWL(tool,diff(range),mean(range))
    %Update the image
    set(tool.handles.I,'CData',im)
    axes(tool.handles.Axes);
    xlim([0 size(I,2)])
    ylim([0 size(I,1)])

    %Update the gridlines
    axes(tool.handles.Axes);
    delete(tool.handles.grid)
    nGrid=7;
    nMinor=4;
    x=linspace(1,size(I,2),nGrid);
    y=linspace(1,size(I,1),nGrid);
    hold on;
    tool.handles.grid=[];
    gColor=[255 38 38]./256;
```

```
            mColor=[255 102 102]./256;
            for i=1:nGrid
                tool.handles.grid(end+1)=plot([.5 size(I,2)-.5],[y(i) y(i)],'-
','LineWidth',1.2,'HitTest','off','Color',gColor);
                tool.handles.grid(end+1)=plot([x(i) x(i)],[.5 size(I,1)-.5],'-
','LineWidth',1.2,'HitTest','off','Color',gColor);
                if i<nGrid
                    xm=linspace(x(i),x(i+1),nMinor+2); xm=xm(2:end-1);
                    ym=linspace(y(i),y(i+1),nMinor+2); ym=ym(2:end-1);
                    for j=1:nMinor
                        tool.handles.grid(end+1)=plot([.5 size(I,2)-.5],[ym(j)
ym(j)],'-r','LineWidth',.9,'HitTest','off','Color',mColor);
                        tool.handles.grid(end+1)=plot([xm(j) xm(j)],[.5 size(I,1)-
.5],'-r','LineWidth',.9,'HitTest','off','Color',mColor);
                    end
                end
            end

tool.handles.grid(end+1)=scatter(.5+size(I,2)/2,.5+size(I,1)/2,'r','filled');
            toggleGrid(tool.handles.Tools.Grid,[],tool)

            %Update the slider
            setupSlider(tool)

            %Show the first slice
            showSlice(tool)


        end

        function I = getImage(tool)
            I=tool.I;
        end

        function handles=getHandles(tool)
            handles=tool.handles;
        end

        function setDisplayRange(tool,range)
            W=diff(range);
            L=mean(range);
            setWL(tool,W,L);
        end

        function range=getDisplayRange(tool)
            range=get(tool.handles.Axes,'Clim');
        end

        function setWindowLevel(tool,W,L)
            setWL(tool,W,L);
        end

        function [W,L] = getWindowLevel(tool)
            range=get(tool.handles.Axes,'Clim');
            W=diff(range);
            L=mean(range);
        end

        function ROI = getCurrentROI(tool)
            CurrentROI=tool.CurrentROI;
            if ~isempty(CurrentROI)
                if isvalid(CurrentROI)
```

```
                    mask = createMask(CurrentROI);
                    im=get(tool.handles.I,'CData');
                    stats=
regionprops(mask,im,'Area','Perimeter','MaxIntensity','MinIntensity','MeanIntensity');
                    stats.STD=std(im(mask));
                    ROI.mask=mask;
                    ROI.stats=stats;
                end
            else
                ROI=[];
            end
        end

        function setCurrentSlice(tool,slice)
            showSlice(tool,slice)
        end

        function slice = getCurrentSlice(tool)
            slice=round(get(tool.handles.Slider,'value'));
        end

    end

    methods (Access = private)

        function addROIhandles(tool,h)
            ROIhandles=tool.ROIhandles;
            ROIhandles{end+1}=h;
            tool.ROIhandles=ROIhandles;
        end

        function multipleScrollWheel(scr,evnt,tools)
            for i=1:length(tools)
                scrollWheel(scr,evnt,tools(i))
            end
        end

        function scrollWheel(scr,evnt,tool)
            %Check to see if the mouse is hovering over the axis
            units=get(tool.handles.fig,'Units');
            set(tool.handles.fig,'Units','Pixels')
            point=get(tool.handles.fig, 'CurrentPoint');
            set(tool.handles.fig,'Units',units)

            units=get(tool.handles.Panels.Large,'Units');
            set(tool.handles.Panels.Large,'Units','Pixels')
            pos_p=get(tool.handles.Panels.Large,'Position');
            set(tool.handles.Panels.Large,'Units',units)

            units=get(tool.handles.Panels.Image,'Units');
            set(tool.handles.Panels.Image,'Units','Pixels')
            pos_a=get(tool.handles.Panels.Image,'Position');
            set(tool.handles.Panels.Image,'Units',units)

            xmin=pos_p(1)+pos_a(1); xmax=xmin+pos_a(3);
            ymin=pos_p(2)+pos_a(2); ymax=ymin+pos_a(4);

            if point(1)>=xmin && point(1)<=xmax && point(2)>=ymin && point(2)<=ymax
                newSlice=get(tool.handles.Slider,'value')-evnt.VerticalScrollCount;
                if newSlice>=1 && newSlice <=size(tool.I,3)
                    set(tool.handles.Slider,'value',newSlice);
                    showSlice(tool)
```

```
                end
            end

        end
        function showSlice(varargin)
            switch nargin
                case 1
                    tool=varargin{1};
                    n=round(get(tool.handles.Slider,'value'));
                case 2
                    tool=varargin{1};
                    n=varargin{2};
                    set(tool.handles.Slider,'value',n);
                otherwise
                    tool=varargin{1};
                    n=round(get(tool.handles.Slider,'value'));
            end

            if n < 1
                n=1;
            end

            if n > size(tool.I,3)
                n=size(tool.I,3);
            end

            set(tool.handles.I,'CData',tool.I(:,:,n))
            set(tool.handles.SliceText,'String',[num2str(n) '/'
num2str(size(tool.I,3))])
            if get(tool.handles.Tools.Hist,'value')
                im=tool.I(:,:,n);
                nelements=hist(im(:),tool.centers);
nelements=nelements./max(nelements);
                set(tool.handles.HistLine,'YData',nelements);
            end

        end

        function setupSlider(tool)
            n=size(tool.I,3);
            if n==1
                set(tool.handles.Slider,'visible','off');
            else
                set(tool.handles.Slider,'visible','on');
                set(tool.handles.Slider,'min',1,'max',size(tool.I,3),'value',1)
                set(tool.handles.Slider,'SliderStep',[1/(size(tool.I,3)-1)
1/(size(tool.I,3)-1)])
                fun=@(hobject,eventdata)showSlice(tool,[],hobject,eventdata);
                set(tool.handles.Slider,'Callback',fun);
            end

        end

        function setWL(tool,W,L)
            set(tool.handles.Axes,'Clim',[L-W/2 L+W/2])
            set(tool.handles.Tools.W,'String',num2str(W));
            set(tool.handles.Tools.L,'String',num2str(L));
            set(tool.handles.HistImageAxes,'Clim',[L-W/2 L+W/2])
            set(tool.handles.Histrange(1),'XData',[L-W/2 L-W/2 L-W/2])
            set(tool.handles.Histrange(2),'XData',[L+W/2 L+W/2 L+W/2])
            set(tool.handles.Histrange(3),'XData',[L L L])
        end
```

```
        function WindowLevel_callback(hobject,evnt,tool)
            range=get(tool.handles.Axes,'Clim');
            Wold=range(2)-range(1); Lold=mean(range);
            W=str2num(get(tool.handles.Tools.W,'String'));
            if isempty(W) || W<=0
                W=Wold;
                set(tool.handles.Tools.W,'String',num2str(W))
            end
            L=str2num(get(tool.handles.Tools.L,'String'));
            if isempty(L)
                L=Lold;
                set(tool.handles.Tools.L,'String',num2str(L))
            end
            setWL(tool,W,L)
        end

        function imageButtonDownFunction(hObject,eventdata,tool)
            bp=get(tool.handles.Axes,'CurrentPoint');
            bp=[bp(1,1) bp(1,2)];
            switch get(tool.handles.fig,'SelectionType')
                case 'normal'   %Adjust window and level
                    CLIM=get(tool.handles.Axes,'Clim');
                    W=CLIM(2)-CLIM(1);
                    L=mean(CLIM);
                    fun=@(src,evnt)
adjustContrastMouse(src,evnt,bp,tool.handles.Axes,tool,W,L);
                    fun2=@(src,evnt) buttonUpFunction(src,evnt,tool);

set(tool.handles.fig,'WindowButtonMotionFcn',fun,'WindowButtonUpFcn',fun2)
                case 'extend'  %Zoom
                    fun=@(src,evnt)
adjustZoomMouse(src,evnt,bp,tool.handles.Axes,tool);
                    fun2=@(src,evnt) buttonUpFunction(src,evnt,tool);

set(tool.handles.fig,'WindowButtonMotionFcn',fun,'WindowButtonUpFcn',fun2)
                case 'alt'
                    xlims=get(tool.handles.Axes,'Xlim');
                    ylims=get(tool.handles.Axes,'Ylim');
                    fun=@(src,evnt)
adjustPanMouse(src,evnt,bp,tool.handles.Axes,xlims,ylims);
                    fun2=@(src,evnt) buttonUpFunction(src,evnt,tool);

set(tool.handles.fig,'WindowButtonMotionFcn',fun,'WindowButtonUpFcn',fun2)
            end
        end

        function histogramButtonDownFunction(hObject,evnt,tool,line)

            switch line
                case 1 %Lower limit of range
                    fun=@(src,evnt)
newLowerRangePosition(src,evnt,tool.handles.HistAxes,tool);
                    fun2=@(src,evnt) buttonUpFunction(src,evnt,tool);

set(tool.handles.fig,'WindowButtonMotionFcn',fun,'WindowButtonUpFcn',fun2)
                case 2 %Upper limt of range
                    fun=@(src,evnt)
newUpperRangePosition(src,evnt,tool.handles.HistAxes,tool);
                    fun2=@(src,evnt) buttonUpFunction(src,evnt,tool);

set(tool.handles.fig,'WindowButtonMotionFcn',fun,'WindowButtonUpFcn',fun2)
```

```
                case 3 %Middle line
                    fun=@(src,evnt)
newLevelRangePosition(src,evnt,tool.handles.HistAxes,tool);
                    fun2=@(src,evnt) buttonUpFunction(src,evnt,tool);

set(tool.handles.fig,'WindowButtonMotionFcn',fun,'WindowButtonUpFcn',fun2)
            end
        end

        function toggleGrid(hObject,eventdata,tool)
            if get(hObject,'Value')
                set(tool.handles.grid,'Visible','on')
            else
                set(tool.handles.grid,'Visible','off')
            end
        end

        function changeColormap(hObject,eventdata,tool)
            n=get(hObject,'Value');
            maps=get(hObject,'String');
            colormap(maps{n})
        end

        function exportROI(hObject,evnt,tool)
            CurrentROI=tool.CurrentROI;
            if ~isempty(CurrentROI)
                if isvalid(CurrentROI)
                    mask = createMask(CurrentROI);
                    im=get(tool.handles.I,'CData');
                    stats=
regionprops(mask,im,'Area','Perimeter','MaxIntensity','MinIntensity','MeanIntensity');
                    stats.STD=std(im(mask));
                    ROI.mask=mask;
                    ROI.stats=stats;
                    name = inputdlg('Enter variable name');
                    name=name{1};
                    assignin('base', name, ROI)
                end
            end
        end

        function measureImageCallback(hObject,evnt,tool,type)

            switch type
                case 'ellipse'
                    fcn = makeConstrainToRectFcn('imellipse',[1 size(tool.I,2)],[1
size(tool.I,1)]);
                    h = imellipse(tool.handles.Axes,'PositionConstraintFcn',fcn);
                    addROIhandles(tool,h)
                    fcn=@(pos) newROIposition(pos,h,tool);
                    addNewPositionCallback(h,fcn);
                    setPosition(h,getPosition(h));
                case 'rectangle'
                    fcn = makeConstrainToRectFcn('imrect',[1 size(tool.I,2)],[1
size(tool.I,1)]);
                    h = imrect(tool.handles.Axes,'PositionConstraintFcn',fcn);
                    addROIhandles(tool,h)
                    fcn=@(pos) newROIposition(pos,h,tool);
                    addNewPositionCallback(h,fcn);
                    setPosition(h,getPosition(h));
                case 'polygon'
```

```
                    fcn = makeConstrainToRectFcn('impoly',[1 size(tool.I,2)],[1
size(tool.I,1)]);
                    h = impoly(tool.handles.Axes,'PositionConstraintFcn',fcn);
                    addROIhandles(tool,h)
                    fcn=@(pos) newROIposition(pos,h,tool);
                    addNewPositionCallback(h,fcn);
                    setPosition(h,getPosition(h));
                case 'ruler'
                    h = imdistline(tool.handles.Axes);
                    fcn = makeConstrainToRectFcn('imline',[1 size(tool.I,2)],[1
size(tool.I,1)]);
                    setPositionConstraintFcn(h,fcn);
                case 'profile'
                    axes(tool.handles.Axes);
                    improfile(); grid on;
                otherwise
            end


        end

        function deleteCurrentROI(hObject,evnt,tool)
            CurrentROI=tool.CurrentROI;
            if length(CurrentROI)>0
                if isvalid(CurrentROI)
                    delete(CurrentROI)
                    set(tool.handles.ROIinfo,'String','STD:                    Mean:
');
                end
            end
        end

        function displayHelp(hObject,evnt,tool)

            message={'Welcome to imtool3D', ...
                '',...
                'Left Mouse Button: Window and Level', ...
                'Right Mouse Button: Pan', ...
                'Middle Mouse Button: Zoom', ...
                'Scroll Wheel: Change Slice',...
                '',...
                'Written by Justin Solomon',...
                'Send questions to justin.solomon@duke.edu'};

            msgbox(message)
        end

        function CropImageCallback(hObject,evnt,tool)
            [I2 rect] = imcrop(tool.handles.Axes);
            rect=round(rect);
            setImage(tool, tool.I(rect(2):rect(2)+rect(4)-1,rect(1):rect(1)+rect(3)-
1,:))

        end

        function resetViewCallback(hObject,evnt,tool)
            set(tool.handles.Axes,'Xlim',get(tool.handles.I,'XData'))
            set(tool.handles.Axes,'Ylim',get(tool.handles.I,'YData'))
        end

    end
```

```
end
function deleteCurrentROI(hObject,evnt,tool)
CurrentROI=tool.CurrentROI;
if length(CurrentROI)>0
    if isvalid(CurrentROI)
        delete(CurrentROI)
        set(tool.handles.ROIinfo,'String','STD:                    Mean:
');
    end
end
end
function exportROI(hObject,evnt,tool)
CurrentROI=tool.CurrentROI;
if ~isempty(CurrentROI)
    if isvalid(CurrentROI)
        mask = createMask(CurrentROI);
        im=get(tool.handles.I,'CData');
        stats=
regionprops(mask,im,'Area','Perimeter','MaxIntensity','MinIntensity','MeanIntensity');
        stats.STD=std(im(mask));
        ROI.mask=mask;
        ROI.stats=stats;
        name = inputdlg('Enter variable name');
        name=name{1};
        assignin('base', name, ROI)
    end
end
end
function CropImageCallback(hObject,evnt,tool)
[I2 rect] = imcrop(tool.handles.Axes);
rect=round(rect);
setImage(tool, tool.I(rect(2):rect(2)+rect(4)-1,rect(1):rect(1)+rect(3)-1,:))
end
function measureImageCallback(hObject,evnt,tool,type)
switch type
    case 'ellipse'
        fcn = makeConstrainToRectFcn('imellipse',[1 size(tool.I,2)],[1
size(tool.I,1)]);
        h = imellipse(tool.handles.Axes,'PositionConstraintFcn',fcn);
        addROIhandles(tool,h)
        fcn=@(pos) newROIposition(pos,h,tool);
        addNewPositionCallback(h,fcn);
        setPosition(h,getPosition(h));
    case 'rectangle'
        fcn = makeConstrainToRectFcn('imrect',[1 size(tool.I,2)],[1 size(tool.I,1)]);
        h = imrect(tool.handles.Axes,'PositionConstraintFcn',fcn);
        addROIhandles(tool,h)
        fcn=@(pos) newROIposition(pos,h,tool);
        addNewPositionCallback(h,fcn);
        setPosition(h,getPosition(h));
    case 'polygon'
        fcn = makeConstrainToRectFcn('impoly',[1 size(tool.I,2)],[1 size(tool.I,1)]);
        h = impoly(tool.handles.Axes,'PositionConstraintFcn',fcn);
        addROIhandles(tool,h)
        fcn=@(pos) newROIposition(pos,h,tool);
        addNewPositionCallback(h,fcn);
        setPosition(h,getPosition(h));
    case 'ruler'
        h = imdistline(tool.handles.Axes);
        fcn = makeConstrainToRectFcn('imline',[1 size(tool.I,2)],[1 size(tool.I,1)]);
        setPositionConstraintFcn(h,fcn);
    case 'profile'
```

```
        axes(tool.handles.Axes);
        improfile(); grid on;
    otherwise
end
end
function imageButtonDownFunction(hObject,eventdata,tool)
bp=get(tool.handles.Axes,'CurrentPoint');
bp=[bp(1,1) bp(1,2)];
switch get(tool.handles.fig,'SelectionType')
    case 'normal'   %Adjust window and level
        CLIM=get(tool.handles.Axes,'Clim');
        W=CLIM(2)-CLIM(1);
        L=mean(CLIM);
        fun=@(src,evnt) adjustContrastMouse(src,evnt,bp,tool.handles.Axes,tool,W,L);
        fun2=@(src,evnt) buttonUpFunction(src,evnt,tool);
        set(tool.handles.fig,'WindowButtonMotionFcn',fun,'WindowButtonUpFcn',fun2)
    case 'extend'  %Zoom
        fun=@(src,evnt) adjustZoomMouse(src,evnt,bp,tool.handles.Axes,tool);
        fun2=@(src,evnt) buttonUpFunction(src,evnt,tool);
        set(tool.handles.fig,'WindowButtonMotionFcn',fun,'WindowButtonUpFcn',fun2)
    case 'alt'
        xlims=get(tool.handles.Axes,'Xlim');
        ylims=get(tool.handles.Axes,'Ylim');
        fun=@(src,evnt) adjustPanMouse(src,evnt,bp,tool.handles.Axes,xlims,ylims);
        fun2=@(src,evnt) buttonUpFunction(src,evnt,tool);
        set(tool.handles.fig,'WindowButtonMotionFcn',fun,'WindowButtonUpFcn',fun2)
end
end
function resetViewCallback(hObject,evnt,tool)
set(tool.handles.Axes,'Xlim',get(tool.handles.I,'XData'))
set(tool.handles.Axes,'Ylim',get(tool.handles.I,'YData'))
end
function toggleGrid(hObject,eventdata,tool)
if get(hObject,'Value')
    set(tool.handles.grid,'Visible','on')
else
    set(tool.handles.grid,'Visible','off')
end
end
function changeColormap(hObject,eventdata,tool)
n=get(hObject,'Value');
maps=get(hObject,'String');
colormap(maps{n})
end
function displayHelp(hObject,evnt,tool)
message={'Welcome to imtool3D', ...
    '',...
    'Left Mouse Button: Window and Level', ...
    'Right Mouse Button: Pan', ...
    'Middle Mouse Button: Zoom', ...
    'Scroll Wheel: Change Slice',...
    '',...
    'Written by Justin Solomon',...
    'Send questions to justin.solomon@duke.edu'};
msgbox(message)
end
function WindowLevel_callback(hobject,evnt,tool)
range=get(tool.handles.Axes,'Clim');
Wold=range(2)-range(1); Lold=mean(range);
W=str2num(get(tool.handles.Tools.W,'String'));
if isempty(W) || W<=0
    W=Wold;
```

```
    set(tool.handles.Tools.W,'String',num2str(W))
end
L=str2num(get(tool.handles.Tools.L,'String'));
if isempty(L)
    L=Lold;
    set(tool.handles.Tools.L,'String',num2str(L))
end
setWL(tool,W,L)
end

function histogramButtonDownFunction(hObject,evnt,tool,line)
switch line
    case 1 %Lower limit of range
        fun=@(src,evnt) newLowerRangePosition(src,evnt,tool.handles.HistAxes,tool);
        fun2=@(src,evnt) buttonUpFunction(src,evnt,tool);
        set(tool.handles.fig,'WindowButtonMotionFcn',fun,'WindowButtonUpFcn',fun2)
    case 2 %Upper limt of range
        fun=@(src,evnt) newUpperRangePosition(src,evnt,tool.handles.HistAxes,tool);
        fun2=@(src,evnt) buttonUpFunction(src,evnt,tool);
        set(tool.handles.fig,'WindowButtonMotionFcn',fun,'WindowButtonUpFcn',fun2)
    case 3 %Middle line
        fun=@(src,evnt) newLevelRangePosition(src,evnt,tool.handles.HistAxes,tool);
        fun2=@(src,evnt) buttonUpFunction(src,evnt,tool);
        set(tool.handles.fig,'WindowButtonMotionFcn',fun,'WindowButtonUpFcn',fun2)
end
end
function addROIhandles(tool,h)
ROIhandles=tool.ROIhandles;
ROIhandles{end+1}=h;
tool.ROIhandles=ROIhandles;
end
function scrollWheel(scr,evnt,tool)
%Check to see if the mouse is hovering over the axis
units=get(tool.handles.fig,'Units');
set(tool.handles.fig,'Units','Pixels')
point=get(tool.handles.fig, 'CurrentPoint');
set(tool.handles.fig,'Units',units)
units=get(tool.handles.Panels.Large,'Units');
set(tool.handles.Panels.Large,'Units','Pixels')
pos_p=get(tool.handles.Panels.Large,'Position');
set(tool.handles.Panels.Large,'Units',units)
units=get(tool.handles.Panels.Image,'Units');
set(tool.handles.Panels.Image,'Units','Pixels')
pos_a=get(tool.handles.Panels.Image,'Position');
set(tool.handles.Panels.Image,'Units',units)
xmin=pos_p(1)+pos_a(1); xmax=xmin+pos_a(3);
ymin=pos_p(2)+pos_a(2); ymax=ymin+pos_a(4);
if point(1)>=xmin && point(1)<=xmax && point(2)>=ymin && point(2)<=ymax
    newSlice=get(tool.handles.Slider,'value')-evnt.VerticalScrollCount;
    if newSlice>=1 && newSlice <=size(tool.I,3)
        set(tool.handles.Slider,'value',newSlice);
        showSlice(tool)
    end
end
end
function multipleScrollWheel(scr,evnt,tools)
for i=1:length(tools)
    scrollWheel(scr,evnt,tools(i))
end
end
function newLowerRangePosition(src,evnt,hObject,tool)
cp = get(hObject,'CurrentPoint'); cp=[cp(1,1) cp(1,2)];
```

```
range=get(tool.handles.Axes,'Clim');
Xlims=get(hObject,'Xlim');
range(1)=cp(1);
W=diff(range);
L=mean(range);
if W>0 && range(1)>=Xlims(1)
    setWL(tool,W,L)
end
end
function newUpperRangePosition(src,evnt,hObject,tool)
cp = get(hObject,'CurrentPoint'); cp=[cp(1,1) cp(1,2)];
range=get(tool.handles.Axes,'Clim');
Xlims=get(hObject,'Xlim');
range(2)=cp(1);
W=diff(range);
L=mean(range);
if W>0 && range(2)<=Xlims(2)
    setWL(tool,W,L)
end
end
function newLevelRangePosition(src,evnt,hObject,tool)
cp = get(hObject,'CurrentPoint'); cp=[cp(1,1) cp(1,2)];
range=get(tool.handles.Axes,'Clim');
Xlims=get(hObject,'Xlim');
L=cp(1);
W=diff(range);
if L>=Xlims(1) && L<=Xlims(2)
    setWL(tool,W,L)
end
end
function newROIposition(pos,hObject,tool)
ROIhandles=tool.ROIhandles;
for i=1:length(ROIhandles)
    if isvalid(ROIhandles{i})
        setColor(ROIhandles{i},'b');
    end
end
setColor(hObject,'r');
mask = createMask(hObject);
im=get(tool.handles.I,'CData');
m=mean(im(mask));
noise=std(im(mask));
set(tool.handles.ROIinfo,'String',['STD:' num2str(noise,'%+.4f') '   Mean:' 
num2str(m,'%+.4f')])
tool.CurrentROI=hObject;
end
function adjustContrastMouse(src,evnt,bp,hObject,tool,W,L)
cp = get(hObject,'CurrentPoint'); cp=[cp(1,1) cp(1,2)];
d=round(cp-bp);
W2=W+d(1); L=L-d(2);
if W2>=1
    W=W2;
end
setWL(tool,W,L)
end
function adjustZoomMouse(src,evnt,bp,hObject,tool)
cp = get(hObject,'CurrentPoint'); cp=[cp(1,1) cp(1,2)];
d=cp(2)-bp(2);
zFactor=.025;
if d>0
    zoom(1+zFactor)
elseif d<0
```

```
    zoom(1-zFactor)
end
fun=@(Newsrc,Newevnt) adjustZoomMouse(Newsrc,Newevnt,cp,tool.handles.Axes,tool);
set(tool.handles.fig,'WindowButtonMotionFcn',fun)
axis fill
end
function adjustPanMouse(src,evnt,bp,hObject,xlims,ylims)
cp = get(hObject,'CurrentPoint'); cp=[cp(1,1) cp(1,2)];
d=(bp-cp)/1.25;
set(hObject,'Xlim',xlims+d(1),'Ylim',ylims+d(2))
end
function buttonUpFunction(src,evnt,tool)
fun=@(src,evnt)getImageInfo(src,evnt,tool);
set(src,'WindowButtonMotionFcn',fun);
end
function getImageInfo(src,evnt,tool)
pos=round(get(tool.handles.Axes,'CurrentPoint'));
pos=pos(1,1:2);
Xlim=get(tool.handles.Axes,'Xlim');
Ylim=get(tool.handles.Axes,'Ylim');
n=round(get(tool.handles.Slider,'value'));
if n==0
    n=1;
end
if pos(1)>0 && pos(1)<=size(tool.I,2) && pos(1)>=Xlim(1) && pos(1) <=Xlim(2) &&
pos(2)>0 && pos(2)<=size(tool.I,1) && pos(2)>=Ylim(1) && pos(2) <=Ylim(2)
    set(tool.handles.Info,'String',['(' num2str(pos(1)) ',' num2str(pos(2)) ') '
num2str(tool.I(pos(2),pos(1),n))])
else
    set(tool.handles.Info,'String','(x,y) val')
end
end
function panelResizeFunction(hObject,events,tool,w,h,wbutt)
units=get(tool.handles.Panels.Large,'Units');
set(tool.handles.Panels.Large,'Units','Pixels')
pos=get(tool.handles.Panels.Large,'Position');
set(tool.handles.Panels.Large,'Units',units)
if get(tool.handles.Tools.Hist,'value')
    set(tool.handles.Panels.Image,'Position',[w w pos(3)-2*w pos(4)-2*w-h])
else
    set(tool.handles.Panels.Image,'Position',[w w pos(3)-2*w pos(4)-2*w])
end
%set(tool.handles.Panels.Image,'Position',[w w pos(3)-2*w pos(4)-2*w])
set(tool.handles.Panels.Hist,'Position',[w pos(4)-w-h pos(3)-2*w h])
set(tool.handles.Panels.Tools,'Position',[0 pos(4)-w pos(3) w])
set(tool.handles.Panels.ROItools,'Position',[pos(3)-w  w w pos(4)-2*w])
set(tool.handles.Panels.Slider,'Position',[0 w w pos(4)-2*w])
set(tool.handles.Panels.Info,'Position',[0 0 pos(3) w])
axis(tool.handles.Axes,'fill');
buff=(w-wbutt)/2;
pos=get(tool.handles.Panels.ROItools,'Position');
set(tool.handles.Tools.Help,'Position',[buff pos(4)-wbutt-buff wbutt wbutt]);
end
function icon = makeToolbarIconFromPNG(filename)
% makeToolbarIconFromPNG  Creates an icon with transparent
%   background from a PNG image.
%   Copyright 2004 The MathWorks, Inc.
%   $Revision: 1.1.8.1 $  $Date: 2004/08/10 01:50:31 $
  % Read image and alpha channel if there is one.
  [icon,map,alpha] = imread(filename);
  % If there's an alpha channel, the transparent values are 0.  For an RGB
  % image the transparent pixels are [0, 0, 0].  Otherwise the background is
```

```
  % cyan for indexed images.
  if (ndims(icon) == 3) % RGB
    idx = 0;
    if ~isempty(alpha)
      mask = alpha == idx;
    else
      mask = icon==idx;
    end

  else % indexed

    % Look through the colormap for the background color.
    for i=1:size(map,1)
      if all(map(i,:) == [0 1 1])
        idx = i;
        break;
      end
    end

    mask = icon==(idx-1); % Zero based.
    icon = ind2rgb(icon,map);

  end

  % Apply the mask.
  icon = im2double(icon);

  for p = 1:3

    tmp = icon(:,:,p);
    if ndims(mask)==3
        tmp(mask(:,:,p))=NaN;
    else
        tmp(mask) = NaN;
    end
    icon(:,:,p) = tmp;

  end
end
function saveImage(hObject,evnt,tool)
cmap = colormap;
switch get(tool.handles.Tools.SaveOptions,'value')
    case 1 %Save just the current slice
        I=get(tool.handles.I,'CData'); lims=get(tool.handles.Axes,'CLim');
        I=gray2ind(mat2gray(I,lims),256);
        [FileName,PathName] =
uiputfile({'*.png';'*.tif';'*.jpg';'*.bmp';'*.gif';'*.hdf'; ...
            '*.jp2';'*.pbm';'*.pcx';'*.pgm'; ...
            '*.pnm';'*.ppm';'*.ras';'*.xwd'},'Save Image');

        if FileName == 0
        else
            imwrite(I,cmap,[PathName FileName])
        end
    case 2
        lims=get(tool.handles.Axes,'CLim');
        [FileName,PathName] = uiputfile({'*.tif'},'Save Image Stack');
        if FileName == 0
        else
            for i=1:size(tool.I,3)
                imwrite(gray2ind(mat2gray(tool.I(:,:,i),lims),256),cmap, [PathName
FileName], 'WriteMode', 'append',  'Compression','none');
```

```
            end
        end
end
end
function ShowHistogram(hObject,evnt,tool,w,h)
set(tool.handles.Panels.Large,'Units','Pixels')
pos=get(tool.handles.Panels.Large,'Position');
set(tool.handles.Panels.Large,'Units','normalized')
if get(tool.handles.Tools.Hist,'value')
    set(tool.handles.Panels.Image,'Position',[w w pos(3)-2*w pos(4)-2*w-h])
else
    set(tool.handles.Panels.Image,'Position',[w w pos(3)-2*w pos(4)-2*w])
end
axis(tool.handles.Axes,'fill');
showSlice(tool)
end
```
