# Supplementary material for: Reliability of two different measuring techniques with computer tomography for penetration and distribution of cement in the proximal tibia after total knee arthroplasty
Source: BMC Musculoskelet Disord. 2020 Jun 12;21:374. doi: 10.1186/s12891-020-03390-3 (PMC7291566; doi:10.1186/s12891-020-03390-3)
Supplement: Supplementary file 1 — Additional file 1. [file 12891_2020_3390_MOESM1_ESM.zip › Matlab SOP defR4.docx]

**Calculating cement penetration and distribution with CT after total knee arthroplasty with Matlab**

This document will explain how to analyze the cement penetration and distribution with the Matlab scripts. The Matlab scripts were built using Matlab R2019a. If an older version of Matlab is used, some functions might not work properly.

The following steps need to be performed **BEFORE** running the CalculateCement.m script for the first time:

1. Save all the Matlab scripts in a single folder.
2. Open Matlab and go to “Set Path” to add the direction of the folder containing the Matlab scripts and to add the folder containing (the folders with) the dicom files to analyse. This last folder should be added with subfolders if each participant has a folder of its own.
3. Adjust the directory in line **76** of Matlab script **postclibrationBaseplate.m** to the directory of the folder containing (the folders with) the dicom files showing the baseplate.

After all the steps above have been performed, run the CalculateCement.m script. Please follow the instruction given by the dialog boxes. For those who wish for a more detailed explanation, please follow the steps below:

1. Run the CalculateCement.m script.
2. Select whether a new Excel file should be made or an existing Excel file should be used. If a new Excel file must be made, type in the name for the file. The name cannot contain spaces and must end with the “.xlsx” extension.
3. Select a directory in which the Excel file should be saved.
4. Select the dicom file containing the baseplate of the patient you wish to analyze.
5. The contour of the projected baseplate should be uninterrupted.
   If this is not the case, close the contour of the baseplate by clicking and dragging the left mouse button along the interrupted contour. Connect the line back to the starting point (looping within and not outside the projected baseplate).
   If the contour is already closed, double click on the left mouse button within the baseplate.
6. Confirm whether the selected contour of the baseplate is closed. If you are not satisfied with the shape of the contour, click ‘No’ in the dialog box and repeat the process.
7. To create a solid surface of the projected baseplate, fill the blue areas within the projected baseplate by clicking once with the left mouse button on each blue area. This will place a marker in the area. Once all areas have been marked, double click on the left mouse button on any area within the baseplate.
   ATTENTION: do not fill the center circle of the baseplate.
8. If the contour of the projected baseplate is not true to its original form (for example due to CT artefacts), it is possible to trim the contour. To do this, hold the left mouse button and draw around the contour to remove the artefacts. Connect the line back to the starting point (looping outside and not within the baseplate for this step). After all trimming is done and the result is satisfying, select ‘Done’ in the dialog box. If more trimming is required, select the option ‘Trim more’.
9. Select the contour of the prosthesis. To do this, hold and drag the left mouse button around the prosthesis. Make sure only the center of the prothesis is selected; any artefacts should not be included. If the result is satisfying, select ‘Yes’ in the dialog box.
10. Read the dialog box. If you wish to keep the instructions visible while performing them, do not click on OK. Otherwise click OK and the dialog box will disappear. Draw a circle aligned with the center of the prosthesis (i.e. the circle that was not filled in step 7) by holding the left mouse button and dragging it. After the circle is drawn it can be moved to the right position. When satisfied, press on the enter button.
    The center of this circle defines the center between left and right of the projected baseplate.
11. Read the dialog box. If you wish to keep the instructions visible while performing them, do not click on OK. Otherwise click OK and the dialog box will disappear. Draw a line between the posterior points of the keel of the prosthesis by holding the left mouse button. After the line is drawn, it is possible that the line is not yet in the proper position between the bottom points of the prosthesis. Therefore, it can be moved to the proper position after being drawn. When satisfied, press on the enter button.
12. Read the dialog box. If you wish to keep the instructions visible while performing them, do not click on OK. Otherwise click OK and the dialog box will disappear. Move the axis with the arrow keys (up/down) so that the horizontal line intersects with the bottom of the prosthesis center. The projected baseplate is now divided into quadrants.
    The axis can be altered by adjusting the green line and/or by adjusting the red circle and pressing ’r’ on the keyboard . When satisfied with the quadrants, press the enter button. A dialog box will show; select ‘Yes’ if satisfied. If not satisfied, select ‘No’ and repeat the process.
13. Select which side is in the scan (left knee or right knee).
14. The script will now calculate the various percentages of cement penetration for each quadrant and show this in a graph. The results are automatically saved in the Excel file.
15. If Matlab is not closed, run the CalculateCement.m script again to continue analyzing a different patient from step 4. If Matlab has been closed, running the CalculateCement.m script will start from step 1 again. Choose the existing Excel file to continue with the previous analysis. If a patient is analyzed twice the save will overwrite the previous results in the Excel file.
