# Supplementary material for: Reliability of two different measuring techniques with computer tomography for penetration and distribution of cement in the proximal tibia after total knee arthroplasty
Source: BMC Musculoskelet Disord. 2020 Jun 12;21:374. doi: 10.1186/s12891-020-03390-3 (PMC7291566; doi:10.1186/s12891-020-03390-3)
Supplement: Supplementary file 1 — Additional file 1. [file 12891_2020_3390_MOESM1_ESM.zip › postcalibration3DIMtoolR4.pdf]

```
%filenamefurther.m Processes DICOM files.
%   Description of what it does
%   Copyright 2010 Cosimo Bonetti, Joris J. Snellenburg
%   Distributed under the terms of the BSD License
%clear all
clc
%added by roman
folder_name = path;
files = dir(folder_name);
kak = [];
layers=3; %hard code number of files
for i=1:layers
    if(files(i).isdir == 1)
        kak = [kak i];
    end
end
files(kak) =[];
for tellertje = 1:layers
filename = [files(tellertje).folder,'\',files(tellertje).name];
%%%%%%%%%%%%%%%%%%%%%%%%%%%%%%%%%%%%%%%%%%%%%%%%%%%%%%%
%%%%%%%%%%%%%%%%%%%%%%%%%%%%%%%%%%%%%%%%%%%%%%%%%%%%%%%
%%%%%%%%%%%%%%%%%%%%%%%%%%%%%%%%%%%%%%%%%%%%%%%%%%%%%%%
%% HU-parameters
% %     Mask0=I2>a  select the region of interest
% %     Trabecular=(I2>a & I2<b);
% %     Cortical=(I2>c & I2<d);
% %     Cement=(I2>e & I2<f);
% %     Prothesis=(I2>g);

a=400;
b=550;
c=1600;
d=2110;
e=550;
f=1600;
g=2110;
% a=200;
% b=216;
% c=1006;
% d=1804;
% e=1502;
% f=2000;
 P=[a,b,c,d,e,f,g];
save('P.mat','P');
%%%%%%%%%%%%%%%%%%%%%%%%%%%%%%%%%%%%%%%%%%%%%%%%%%%%%%%
%%%%%%%%%%%%%%%%%%%%%%%%%%%%%%%%%%%%%%%%%%%%%%%%%%%%%%%
%%%%%%%%%%%%%%%%%%%%%%%%%%%%%%%%%%%%%%%%%%%%%%%%%%%%%%%
%% Select Images
% If you want to check all the images set "ALLES" TRUE,
ALLES=false;
% If you want to check the Recalibrated slice  set "REC" TRUE,
REC=true;
% If you want to check the Segmentation  set "SEG" TRUE,
SEG=true;
% If you want to check the Trabecular segmentation  set "TRAB" TRUE,
TRAB=false;

% If you want to check the Cortical segmentation  set "CORT" TRUE,
CORT=true;

% If you want to check the Cement segmentation  set "CEM" TRUE,
CEM=true;
```

```
% If you want to check the Prothesis segmentation  set "PROTH" TRUE,
PROTH=false;
%%%%%%%%%%%%%%%%%%%%%%%%%%%%%%%%%%%%%%%%%%%%%%%%%%%%%%%
%%%%%%%%%%%%%%%%%%%%%%%%%%%%%%%%%%%%%%%%%%%%%%%%%%%%%%%
%%%%%%%%%%%%%%%%%%%%%%%%%%%%%%%%%%%%%%%%%%%%%%%%%%%%%%%
%% Load dicom file
fprintf('Starting program ...\n');
% filename= uigetfile('*', 'Select DICOM  for Post-Calibration');
% if isequal(filename,0)
%    disp('No DICOM files (*.dcm) were selected');
% else
%    disp(['DICOM (*.dcm) filenames:...      ', filename])
%    fprintf('Processing DICOM files, ...\n');
% end
%Start the timer
tStart = tic;
%% Post-Calibration
    I=dicomread(filename);
    meta=dicominfo(filename);
    I2=int16(I).*double(meta.RescaleSlope)+double(meta.RescaleIntercept);

    Mask0=I2>P(1); %segmentation threshould; all pixels with HU>P(1)


    [Labeled,numObjects] = bwlabel(Mask0,4); %label segmented area's
    STATS = regionprops(Labeled, I2, 'MajorAxisLength');


    %-------------------------remove the table-------------------------%
    for j=1:numObjects
        if (STATS(j).MajorAxisLength>500)
            pixels=find(Labeled==j);
            Mask0(pixels)=0;
            clear pixels
        end
    end

    %-------------------------Trabecular Bone-------------------------%

    Trabecular=(I2>P(1) & I2<P(2));
    Trabecular=Trabecular.*Mask0; % remove linear structures in Trabecular



    %-------------------------Cortical Bone---------  ----------------%

    Cortical=(I2>P(3) & I2<P(4));
    Cortical=Cortical.*Mask0; % remove linear structures in Cortical


    %----------------------Penetrated Cement Bone----------------------%

    Cement=(I2>P(5) & I2<P(6));
    Cement=Cement.*Mask0; % remove linear structures in Pentrated Cement


    %-------------------------Prothesis-------------------------%
    Prothesis=(I2>P(7));
    Prothesis=Prothesis.*Mask0; % remove linear structures in Prothesis
    if tellertje==1
        figure
```

```
        image(Prothesis,'CDataMapping','scaled')
        approved=0;
        while approved==0
            close all
            image(Prothesis,'CDataMapping','scaled')
            set(gcf,'Position',[800 250 480 400],'Name', 'Select prothesis center')
            contour= images.roi.AssistedFreehand('LineWidth',3);
            keypress = 0;
            draw(contour)
            closed=createMask(contour);
            ProthesisNew=Prothesis.*closed;
            image(ProthesisNew,'CDataMapping','scaled')

            happy = questdlg('Are you satisfied with this contour?',...
                'Check',...
                'Yes','No','Yes');

            switch happy
                case 'No'
                    approved = 0;
                case 'Yes'
                    approved = 1;
            end
        end
        Prothesis=ProthesisNew;
    end
%-------------------------AREA-------------------------%
    %
    %
    %-------------------------VOXEL-------------------------%
    H=double(meta.SliceThickness);
    LD=double(meta.PixelSpacing);
    L=LD(1,:);
    D=LD(2,:);
    Vox=(H*L*D)/10^3;


    %
    %
    %-------------------------Total Volume-------------------------%
    TotSegArea=length(find(Mask0));
    TotVol=TotSegArea*Vox;


    %
    %
    %-------------------------Trabecular Volume-------------------------%
    TrabecularSegArea=length(find(Trabecular));
    TrabecularVol=TrabecularSegArea*Vox;
    %
    %
    %-------------------------Penetrated Cement Volume-------------------------%
    CementSegArea=length(find(Cement));
    CementVol=CementSegArea*Vox;


    %
    %
    %-------------------------Cortical Volume-------------------------%
    CorticalSegArea=length(find(Cortical));
    CorticalVol=CorticalSegArea*Vox;
```

```
    %
    %
    %------------------------Prothesis Volume------------------------%
    ProthesisSegArea=length(find(Prothesis));
    ProthesisVol=ProthesisSegArea*Vox;

    %
    %
    %-----------------------------Ratio------------------------------%
    TrabecularRatio=TrabecularSegArea*100/TotSegArea;
    CorticalRatio=CorticalSegArea*100/TotSegArea;
    CementRatio=CementSegArea*100/TotSegArea;
    ProthesisRatio=ProthesisSegArea*100/TotSegArea;

    drieDcement(:,:,tellertje)=Cement;
    exCem = logical(Cement) & ~logical(basePlate);
    forUse(:,:,tellertje)=(basePlate+Cement-Prothesis).*basePlate+3.*exCem;
    patNum=str2double(files(1).name(end-4));

    %% get patient number
    B = regexp(files(1).name,'\d*','Match');
    for ii= 1:length(B)
        if ~isempty(B{ii})
            Num(ii,1)=str2double(B{ii});
        else
            Num(ii,1)=NaN;
        end
    end
    patNum=Num(end);
    imtool close all
end
%
%     if(ALLES)
%         imtool(I2);
%         imtool(Mask0)
%         imtool(Labeled);
%         imtool(Trabecular)
%         imtool(Cortical)
%         imtool(Cement)
%         imtool(Prothesis)
%     end
%
%     if(REC)
%         imtool(I2);
%     end
%
%     if(SEG)
%         imtool(Mask0)
%     end
%
%     if(TRAB)
%         imtool(Trabecular)
%     end
%
%     if(CORT)
%         imtool(Cortical)
%     end
%
%     if(CEM)
%         imtool(Cement)
%     end
%
```

```
%     if(PROTH)
%         imtool(Prothesis)
%     end
%
%
%     ImageParameters.filename=filename;
%     ImageParameters.VoxelVolume_in_cm3=Vox;
%     ImageParameters.TotalArea_in_pixel=TotSegArea;
%     ImageParameters.TotalVOL_in_cm3=TotVol;
%     ImageParameters.TrabecularArea_in_pixel=TrabecularSegArea;
%     ImageParameters.TrabecularVOL_in_cm3=TrabecularVol;
%     ImageParameters.PenetrtedCementArea_in_pixel=CementSegArea;
%     ImageParameters.PenetrtedCementVOL_in_cm3=CementVol;
%     ImageParameters.CorticalArea_in_pixel=CorticalSegArea;
%     ImageParameters.CorticalVOL_in_cm3=CorticalVol;
%     ImageParameters.ProthesisArea_in_pixel=ProthesisSegArea;
%     ImageParameters.PenetrtedCementVOL_in_cm3=ProthesisVol;
%     ImageParameters.TrabecularArea_percentage=TrabecularRatio;
%     ImageParameters.CorticalArea_percentage=CorticalRatio;
%     ImageParameters.CementArea_percentage=CementRatio;
%     ImageParameters.ProthesisArea_percentage=ProthesisRatio;
%
%     display(ImageParameters)
%
%
%     P1=[double(meta.BitsAllocated),double(meta.Rows),double(meta.Columns),L,D,H];
%     PatientID=meta.PatientID;
%
%     save('P1.mat','P1');
%     save('PatientID.mat','PatientID');
% %
% %
% %     str=['Voxel Volume=', num2str(Vox), 'cm^3']
% %     str1=['TotalArea=', num2str(TotSegArea), 'pixels']
% %     str1a=['TotalVOL=', num2str(TotVol), 'cm^3']
% %     str2=['TrabecularArea=', num2str(TrabecularSegArea), 'pixels']
% %     str2a=['TrabecularVOL=', num2str(TrabecularVol), 'cm^3']
% %     str3=['PenetrtedCementArea=', num2str(CementSegArea), 'pixels']
% %     str3a=['PenetrtedCementVOL=', num2str(CementVol), 'cm^3']
% %     str3=['CorticalArea=', num2str(CorticalSegArea), 'pixels']
% %     str3a=['CorticalVOL=', num2str(CorticalVol), 'cm^3']
% %     str4=['ProthesisArea=', num2str(ProthesisSegArea), 'pixels']
% %     str4a=['PenetrtedCementVOL=', num2str(ProthesisVol), 'cm^3']
% %     str5a=strcat('TrabecularArea percentage=', num2str(TrabecularRatio), '%')
% %     str6a=strcat('CorticalArea percentage=', num2str(CorticalRatio), '%')
% %     str7a=strcat('CementArea percentage=', num2str(CementRatio), '%')
% %     str8a=strcat('ProthesisArea percentage=', num2str(ProthesisRatio), '%')
```
