# Supplementary material for: Reliability of two different measuring techniques with computer tomography for penetration and distribution of cement in the proximal tibia after total knee arthroplasty
Source: BMC Musculoskelet Disord. 2020 Jun 12;21:374. doi: 10.1186/s12891-020-03390-3 (PMC7291566; doi:10.1186/s12891-020-03390-3)
Supplement: Supplementary file 1 — Additional file 1. [file 12891_2020_3390_MOESM1_ESM.zip › postcalibrationBaseplateR4.pdf]

```matlab
%filenamefurther.m Processes DICOM files.
%   Description of what it does
%   Copyright 2010 Cosimo Bonetti, Joris J. Snellenburg
%   Distributed under the terms of the BSD License
%clear all
clc
%%%%%%%%%%%%%%%%%%%%%%%%%%%%%%%%%%%%%%%%%%%%%%%%%%%%%%%
%%%%%%%%%%%%%%%%%%%%%%%%%%%%%%%%%%%%%%%%%%%%%%%%%%%%%%%
%%%%%%%%%%%%%%%%%%%%%%%%%%%%%%%%%%%%%%%%%%%%%%%%%%%%%%%
%% HU-parameters
% %     Mask0=I2>a  select the region of interest
% %     Trabecular=(I2>a & I2<b);
% %     Cortical=(I2>c & I2<d);
% %     Cement=(I2>e & I2<f);
% %     Prothesis=(I2>g);

a=400; %was 192
b=550;
c=1600;
d=2110;
e=550;
f=1600;
g=2110;
% a=200;
% b=216;
% c=1006;
% d=1804;
% e=1502;
% f=2000;
 P=[a,b,c,d,e,f,g];
save('P.mat','P');
%%%%%%%%%%%%%%%%%%%%%%%%%%%%%%%%%%%%%%%%%%%%%%%%%%%%%%%
%%%%%%%%%%%%%%%%%%%%%%%%%%%%%%%%%%%%%%%%%%%%%%%%%%%%%%%
%%%%%%%%%%%%%%%%%%%%%%%%%%%%%%%%%%%%%%%%%%%%%%%%%%%%%%%
%% Select Images
% If you want to check all the images set "ALLES" TRUE,
ALLES=false;
% If you want to check the Recalibrated slice  set "REC" TRUE,
REC=false;
% If you want to check the Segmentation  set "SEG" TRUE,
SEG=true;
% If you want to check the Trabecular segmentation  set "TRAB" TRUE,
TRAB=false;

% If you want to check the Cortical segmentation  set "CORT" TRUE,
CORT=false;

% If you want to check the Cement segmentation  set "CEM" TRUE,
CEM=false;

% If you want to check the Prothesis segmentation  set "PROTH" TRUE,
PROTH=true;
%%%%%%%%%%%%%%%%%%%%%%%%%%%%%%%%%%%%%%%%%%%%%%%%%%%%%%%
%%%%%%%%%%%%%%%%%%%%%%%%%%%%%%%%%%%%%%%%%%%%%%%%%%%%%%%
%%%%%%%%%%%%%%%%%%%%%%%%%%%%%%%%%%%%%%%%%%%%%%%%%%%%%%%
%% Load dicom file
fprintf('Starting program ...\n');
[filename,path]= uigetfile('*','Select DICOM  for Post-
Calibration','C:\Users\Onderzoek Orthopedie\Documents\TKA studie hennie\CT-scans\');
filename=[path,filename]
if isequal(filename,0)
   disp('No DICOM files (*.dcm) were selected');
```

```
else
   disp(['DICOM (*.dcm) filenames:...      ', filename])
   fprintf('Processing DICOM files, ...\n');
end
%Start the timer
tStart = tic;
%% Post-Calibration
    I=dicomread(filename);
    meta=dicominfo(filename);
    I2=int16(I).*double(meta.RescaleSlope)+double(meta.RescaleIntercept);

    Mask0=I2>P(1); %segmentation threshould; all pixels with HU>P(1)


    [Labeled,numObjects] = bwlabel(Mask0,4); %label segmented area's
    STATS = regionprops(Labeled, I2, 'MajorAxisLength');


    %-------------------------remove the table-------------------------%
    for j=1:numObjects
        if (STATS(j).MajorAxisLength>500)
            pixels=find(Labeled==j);
            Mask0(pixels)=0;
            clear pixels
        end
    end

    %-------------------------Trabecular Bone-------------------------%

    Trabecular=(I2>P(1) & I2<P(2));
    Trabecular=Trabecular.*Mask0; % remove linear structures in Trabecular



    %-------------------------Cortical Bone---------  ----------------%

    Cortical=(I2>P(3) & I2<P(4));
    Cortical=Cortical.*Mask0; % remove linear structures in Cortical


    %----------------------Penetrated Cement Bone----------------------%

    Cement=(I2>P(5) & I2<P(6));
    Cement=Cement.*Mask0; % remove linear structures in Pentrated Cement


    %-------------------------Prothesis-------------------------%
    Prothesis=(I2>P(7));
    Prothesis=Prothesis.*Mask0; % remove linear structures in Prothesis
 %-------------------------AREA-------------------------%
    %
    %
    %-------------------------VOXEL-------------------------%
    H=double(meta.SliceThickness);
    LD=double(meta.PixelSpacing);
    L=LD(1,:);
    D=LD(2,:);
    Vox=(H*L*D)/10^3;


    %
    %
```

```
%-------------------------Total Volume-------------------------%
TotSegArea=length(find(Mask0));
TotVol=TotSegArea*Vox;


%
%
%-------------------------Trabecular Volume-------------------------%
TrabecularSegArea=length(find(Trabecular));
TrabecularVol=TrabecularSegArea*Vox;
%
%
%-------------------------Penetrated Cement Volume-------------------------%
CementSegArea=length(find(Cement));
CementVol=CementSegArea*Vox;


%
%
%-------------------------Cortical Volume-------------------------%
CorticalSegArea=length(find(Cortical));
CorticalVol=CorticalSegArea*Vox;

%
%
%-------------------------Prothesis Volume-------------------------%
ProthesisSegArea=length(find(Prothesis));
ProthesisVol=ProthesisSegArea*Vox;

%
%
%------------------------------Ratio------------------------------%
TrabecularRatio=TrabecularSegArea*100/TotSegArea;
CorticalRatio=CorticalSegArea*100/TotSegArea;
CementRatio=CementSegArea*100/TotSegArea;
ProthesisRatio=ProthesisSegArea*100/TotSegArea;



if(ALLES)
    imtool(I2);
    imtool(Mask0)
    imtool(Labeled);
    imtool(Trabecular)
    imtool(Cortical)
    imtool(Cement)
    imtool(Prothesis)
end

if(REC)
    imtool(I2);
end

if(SEG)
    imtool(Mask0)
end

if(TRAB)
    imtool(Trabecular)
end

if(CORT)
```

```
    imtool(Cortical)
end

if(CEM)
    imtool(Cement)
end

approved=0;
if(PROTH)
    area=500;
    close all
    while approved==0
        image(Prothesis,'CDataMapping','scaled')
        set(gcf,'Position',get(0,'Screensize'),'Name', 'Close baseplate contour')
        contour= images.roi.AssistedFreehand('LineWidth',1);
        draw(contour)
        closed=createMask(contour);
        ProthesisNew=Prothesis+closed-closed.*(Prothesis);
        image(ProthesisNew,'CDataMapping','scaled')

        happy = questdlg('Is the contour of the baseplate closed?',...
            'Check',...
            'Yes','No','Yes');

        switch happy
            case 'No'
                approved = 0;
            case 'Yes'
                approved = 1;
        end
    end
    Prothesis=ProthesisNew;
    logProth=logical(Prothesis);
    logProth=imfill(logProth);
    filled=imfill(logProth,'holes');
    bpFull=imfill(double(logProth));
    holes= filled & ~logProth;
    bigholes=bwareaopen(holes,area);
    smallholes=holes & ~bigholes;
    new=logProth | smallholes;
    inv=~new;
    filled=imfill(inv,'holes');
    holes= filled & ~inv;
    bigholes=bwareaopen(holes,area);
    smallholes=holes & ~bigholes;
    inv=inv | smallholes;
    new=~inv;
    figure
    approved=0;
    while approved==0

        image(new,'CDataMapping','scaled')
        set(gcf,'Position',get(0,'Screensize'),'Name', 'Trim excess baseplate')
        contour= images.roi.AssistedFreehand('LineWidth',1);
        draw(contour)
        closed=createMask(contour);
        ProthesisNew=new-closed.*new;
        image(ProthesisNew,'CDataMapping','scaled')

        happy = questdlg('Are you satisfied with this contour?',...
            'Check',...
            'Back','Trim more','Done','Done');
```

```
        switch happy
            case 'Back'
                approved = 0;
            case 'Trim more'
                approved = 0;
                new=ProthesisNew;
            case 'Done'
                approved = 1;
        end
    end
    basePlate=ProthesisNew;

end

ImageParameters.filename=filename;
ImageParameters.VoxelVolume_in_cm3=Vox;
ImageParameters.TotalArea_in_pixel=TotSegArea;
ImageParameters.TotalVOL_in_cm3=TotVol;
ImageParameters.TrabecularArea_in_pixel=TrabecularSegArea;
ImageParameters.TrabecularVOL_in_cm3=TrabecularVol;
ImageParameters.PenetrtedCementArea_in_pixel=CementSegArea;
ImageParameters.PenetrtedCementVOL_in_cm3=CementVol;
ImageParameters.CorticalArea_in_pixel=CorticalSegArea;
ImageParameters.CorticalVOL_in_cm3=CorticalVol;
ImageParameters.ProthesisArea_in_pixel=ProthesisSegArea;
ImageParameters.PenetrtedCementVOL_in_cm3=ProthesisVol;
ImageParameters.TrabecularArea_percentage=TrabecularRatio;
ImageParameters.CorticalArea_percentage=CorticalRatio;
ImageParameters.CementArea_percentage=CementRatio;
ImageParameters.ProthesisArea_percentage=ProthesisRatio;

display(ImageParameters)
P1=[double(meta.BitsAllocated),double(meta.Rows),double(meta.Columns),L,D,H];
PatientID=meta.PatientID;

save('P1.mat','P1');
save('PatientID.mat','PatientID');

%
%
%     str=['Voxel Volume=', num2str(Vox), 'cm^3']
%     str1=['TotalArea=', num2str(TotSegArea), 'pixels']
%     str1a=['TotalVOL=', num2str(TotVol), 'cm^3']
%     str2=['TrabecularArea=', num2str(TrabecularSegArea), 'pixels']
%     str2a=['TrabecularVOL=', num2str(TrabecularVol), 'cm^3']
%     str3=['PenetrtedCementArea=', num2str(CementSegArea), 'pixels']
%     str3a=['PenetrtedCementVOL=', num2str(CementVol), 'cm^3']
%     str3=['CorticalArea=', num2str(CorticalSegArea), 'pixels']
%     str3a=['CorticalVOL=', num2str(CorticalVol), 'cm^3']
%     str4=['ProthesisArea=', num2str(ProthesisSegArea), 'pixels']
%     str4a=['PenetrtedCementVOL=', num2str(ProthesisVol), 'cm^3']
%     str5a=strcat('TrabecularArea percentage=', num2str(TrabecularRatio), '%')
%     str6a=strcat('CorticalArea percentage=', num2str(CorticalRatio), '%')
%     str7a=strcat('CementArea percentage=', num2str(CementRatio), '%')
%     str8a=strcat('ProthesisArea percentage=', num2str(ProthesisRatio), '%')
```
