# Supplementary material for: Reliability of two different measuring techniques with computer tomography for penetration and distribution of cement in the proximal tibia after total knee arthroplasty
Source: BMC Musculoskelet Disord. 2020 Jun 12;21:374. doi: 10.1186/s12891-020-03390-3 (PMC7291566; doi:10.1186/s12891-020-03390-3)
Supplement: Supplementary file 1 — Additional file 1. [file 12891_2020_3390_MOESM1_ESM.zip › ROIcontourR4.pdf]

```
close all
[contour,handle] = imcontour(bpFull,[1 1], 'r')
newcontour=[];
for i=1:length(contour)
    if contour(1,i)>10
        newcontour=[newcontour contour(:,i)];
    end
end
contour=newcontour;
image(bpFull,'CDataMapping','scaled')
contRoi = images.roi.Freehand(gca,'Position',contour');
cont=contRoi.Position;
newcontour=[];
        for i=1:length(cont)-1
            if abs(cont(i,1)-cont(i+1,1))<2 && abs(cont(i,2)-cont(i+1,2))<2
                newcontour=[newcontour; cont(i,:)];
            end
        end

        contRoi.Position=newcontour


   savedCont=[269.1915  187.4585
  269.0000  188.0000
  270.0000  188.0000
  271.0000  188.0000
  272.0000  188.0000
  272.0000  188.0000
  273.0000  189.0000
  274.0000  189.0000
  275.0000  189.0000
  276.0000  189.0000
  277.0000  189.0000
  278.0000  188.0000
  278.0000  188.0000
  279.0000  188.0000
  280.0000  188.0000
  281.0000  188.0000
  282.0000  188.0000
  283.0000  188.0000
  284.0000  188.0000
  285.0000  188.0000
  286.0000  188.0000
  287.0000  188.0000
  287.0000  188.0000
  288.0000  189.0000
  289.0000  189.0000
  290.0000  189.0000
  291.0000  189.0000
  292.0000  189.0000
  293.0000  189.0000
  294.0000  189.0000
  295.0000  189.0000
  296.0000  189.0000
  297.0000  189.0000
  298.0000  189.0000
  299.0000  189.0000
  300.0000  189.0000
  300.0000  189.0000
  301.0000  190.0000
  302.0000  190.0000
  303.0000  190.0000
```

304.0000	190.0000
305.0000	190.0000
305.0000	190.0000
306.0000	191.0000
307.0000	191.0000
308.0000	191.0000
309.0000	191.0000
309.0000	191.0000
310.0000	192.0000
311.0000	192.0000
312.0000	192.0000
313.0000	192.0000
313.0000	192.0000
314.0000	193.0000
315.0000	193.0000
316.0000	193.0000
316.0000	193.0000
317.0000	194.0000
318.0000	194.0000
319.0000	194.0000
319.0000	194.0000
320.0000	195.0000
321.0000	195.0000
322.0000	195.0000
322.0000	195.0000
323.0000	196.0000
324.0000	196.0000
324.0000	196.0000
325.0000	197.0000
326.0000	197.0000
326.0000	197.0000
327.0000	198.0000
328.0000	198.0000
328.0000	198.0000
329.0000	199.0000
330.0000	199.0000
330.0000	199.0000
331.0000	200.0000
332.0000	200.0000
332.0000	200.0000
333.0000	201.0000
334.0000	201.0000
334.0000	201.0000
335.0000	202.0000
335.0000	202.0000
336.0000	203.0000
337.0000	203.0000
337.0000	203.0000
338.0000	204.0000
338.0000	204.0000
339.0000	205.0000
339.0000	205.0000
340.0000	206.0000
341.0000	206.0000
341.0000	206.0000
342.0000	207.0000
342.0000	207.0000
343.0000	208.0000
343.0000	208.0000
344.0000	209.0000
344.0000	209.0000
345.0000	210.0000

345.0000	210.0000
346.0000	211.0000
346.0000	211.0000
347.0000	212.0000
347.0000	212.0000
348.0000	213.0000
348.0000	213.0000
349.0000	214.0000
349.0000	214.0000
350.0000	215.0000
350.0000	215.0000
351.0000	216.0000
351.0000	216.0000
352.0000	217.0000
352.0000	217.0000
353.0000	218.0000
353.0000	218.0000
353.0000	219.0000
354.0000	220.0000
354.0000	220.0000
355.0000	221.0000
355.0000	221.0000
356.0000	222.0000
356.0000	222.0000
356.0000	223.0000
357.0000	224.0000
357.0000	224.0000
357.0000	225.0000
358.0000	226.0000
358.0000	226.0000
359.0000	227.0000
359.0000	227.0000
359.0000	228.0000
360.0000	229.0000
360.0000	229.0000
360.0000	230.0000
361.0000	231.0000
361.0000	231.0000
362.0000	232.0000
362.0000	232.0000
362.0000	233.0000
363.0000	234.0000
363.0000	234.0000
363.0000	235.0000
364.0000	236.0000
364.0000	236.0000
364.0000	237.0000
365.0000	238.0000
365.0000	238.0000
365.0000	239.0000
365.0000	240.0000
366.0000	241.0000
366.0000	241.0000
366.0000	242.0000
367.0000	243.0000
367.0000	243.0000
367.0000	244.0000
367.0000	245.0000
368.0000	246.0000
368.0000	246.0000
368.0000	247.0000
368.0000	248.0000

368.0000	249.0000
369.0000	250.0000
369.0000	250.0000
369.0000	251.0000
369.0000	252.0000
369.0000	253.0000
370.0000	254.0000
370.0000	254.0000
370.0000	255.0000
370.0000	256.0000
370.0000	257.0000
370.0000	258.0000
370.0000	259.0000
371.0000	260.0000
371.0000	260.0000
371.0000	261.0000
371.0000	262.0000
371.0000	263.0000
371.0000	264.0000
371.0000	265.0000
372.0000	266.0000
372.0000	266.0000
372.0000	267.0000
372.0000	268.0000
372.0000	269.0000
372.0000	270.0000
372.0000	271.0000
372.0000	272.0000
372.0000	273.0000
373.0000	274.0000
373.0000	274.0000
373.0000	275.0000
373.0000	276.0000
373.0000	277.0000
373.0000	278.0000
373.0000	279.0000
373.0000	280.0000
374.0000	281.0000
374.0000	281.0000
374.0000	282.0000
374.0000	283.0000
374.0000	284.0000
374.0000	285.0000
375.0000	286.0000
375.0000	286.0000
375.0000	287.0000
375.0000	287.0000
374.0000	288.0000
375.0000	289.0000
375.0000	289.0000
375.0000	290.0000
375.0000	291.0000
375.0000	292.0000
375.0000	293.0000
375.0000	293.0000
374.0000	294.0000
374.0000	295.0000
374.0000	296.0000
374.0000	297.0000
374.0000	298.0000
374.0000	299.0000
374.0000	300.0000

374.0000	301.0000
374.0000	301.0000
373.0000	302.0000
373.0000	303.0000
373.0000	304.0000
373.0000	304.0000
372.0000	305.0000
372.0000	306.0000
372.0000	306.0000
371.0000	307.0000
371.0000	308.0000
371.0000	308.0000
370.0000	309.0000
370.0000	310.0000
370.0000	310.0000
369.0000	311.0000
369.0000	312.0000
369.0000	312.0000
368.0000	313.0000
368.0000	313.0000
367.0000	314.0000
367.0000	315.0000
367.0000	315.0000
366.0000	316.0000
366.0000	316.0000
365.0000	317.0000
365.0000	317.0000
364.0000	318.0000
364.0000	318.0000
363.0000	319.0000
363.0000	319.0000
362.0000	320.0000
362.0000	320.0000
361.0000	321.0000
361.0000	321.0000
360.0000	322.0000
360.0000	322.0000
359.0000	322.0000
358.0000	323.0000
358.0000	323.0000
357.0000	324.0000
357.0000	324.0000
356.0000	324.0000
355.0000	325.0000
355.0000	325.0000
354.0000	325.0000
353.0000	326.0000
353.0000	326.0000
352.0000	326.0000
351.0000	327.0000
351.0000	327.0000
350.0000	327.0000
349.0000	327.0000
348.0000	328.0000
348.0000	328.0000
347.0000	328.0000
346.0000	328.0000
345.0000	329.0000
345.0000	329.0000
344.0000	329.0000
343.0000	329.0000
342.0000	329.0000

341.0000	330.0000
341.0000	330.0000
340.0000	330.0000
339.0000	330.0000
338.0000	330.0000
337.0000	330.0000
336.0000	330.0000
335.0000	331.0000
335.0000	331.0000
334.0000	331.0000
333.0000	331.0000
332.0000	331.0000
331.0000	331.0000
330.0000	331.0000
329.0000	331.0000
328.0000	332.0000
328.0000	332.0000
327.0000	332.0000
326.0000	332.0000
325.0000	332.0000
324.0000	332.0000
323.0000	332.0000
322.0000	333.0000
322.0000	333.0000
321.0000	333.0000
320.0000	333.0000
319.0000	333.0000
318.0000	333.0000
317.0000	333.0000
316.0000	333.0000
315.0000	334.0000
315.0000	334.0000
314.0000	334.0000
313.0000	334.0000
312.0000	334.0000
311.0000	334.0000
310.0000	334.0000
309.0000	334.0000
308.0000	334.0000
307.0000	334.0000
306.0000	334.0000
305.0000	334.0000
305.0000	334.0000
304.0000	333.0000
303.0000	333.0000
302.0000	333.0000
302.0000	333.0000
301.0000	332.0000
301.0000	332.0000
300.0000	331.0000
300.0000	331.0000
299.0000	330.0000
299.0000	330.0000
298.0000	329.0000
298.0000	329.0000
298.0000	328.0000
297.0000	327.0000
297.0000	327.0000
297.0000	326.0000
296.0000	325.0000
296.0000	325.0000
296.0000	324.0000

295.0000	323.0000
295.0000	323.0000
294.0000	322.0000
294.0000	322.0000
294.0000	321.0000
294.0000	320.0000
294.0000	319.0000
294.0000	318.0000
293.0000	317.0000
293.0000	317.0000
293.0000	316.0000
292.0000	315.0000
292.0000	315.0000
292.0000	314.0000
292.0000	313.0000
291.0000	312.0000
291.0000	312.0000
291.0000	311.0000
290.0000	310.0000
290.0000	310.0000
289.0000	309.0000
289.0000	309.0000
289.0000	308.0000
289.0000	307.0000
289.0000	306.0000
288.0000	305.0000
288.0000	305.0000
287.0000	304.0000
287.0000	304.0000
287.0000	303.0000
287.0000	302.0000
286.0000	301.0000
286.0000	301.0000
286.0000	300.0000
285.0000	299.0000
285.0000	299.0000
285.0000	298.0000
284.0000	297.0000
284.0000	297.0000
283.0000	296.0000
283.0000	296.0000
282.0000	295.0000
281.0000	295.0000
281.0000	295.0000
281.0000	294.0000
280.0000	293.0000
279.0000	293.0000
278.0000	293.0000
278.0000	293.0000
277.0000	292.0000
277.0000	292.0000
276.0000	291.0000
275.0000	291.0000
275.0000	291.0000
274.0000	290.0000
273.0000	290.0000
272.0000	290.0000
271.0000	290.0000
270.0000	290.0000
270.0000	290.0000
269.0000	289.0000
268.0000	289.0000

267.0000	289.0000
266.0000	289.0000
265.0000	289.0000
264.0000	289.0000
263.0000	289.0000
263.0000	289.0000
262.0000	288.0000
261.0000	288.0000
260.0000	288.0000
260.0000	288.0000
259.0000	287.0000
259.0000	287.0000
258.0000	286.0000
257.0000	286.0000
256.0000	286.0000
255.0000	287.0000
255.0000	287.0000
254.0000	287.0000
253.0000	288.0000
253.0000	289.0000
254.0000	290.0000
254.0000	290.0000
255.0000	291.0000
255.0000	291.0000
255.0000	291.0000
254.0000	292.0000
254.0000	292.0000
253.0000	292.0000
252.0000	292.0000
251.0000	292.0000
250.0000	293.0000
250.0000	293.0000
249.0000	294.0000
249.0000	295.0000
249.0000	295.0000
248.0000	296.0000
248.0000	296.0000
247.0000	296.0000
246.0000	297.0000
246.0000	297.0000
245.0000	298.0000
245.0000	298.0000
244.0000	299.0000
244.0000	299.0000
243.0000	300.0000
243.0000	300.0000
242.0000	301.0000
242.0000	301.0000
241.0000	302.0000
241.0000	303.0000
241.0000	304.0000
241.0000	304.0000
240.0000	305.0000
240.0000	306.0000
240.0000	307.0000
240.0000	308.0000
240.0000	309.0000
240.0000	309.0000
239.0000	310.0000
239.0000	311.0000
239.0000	312.0000
239.0000	313.0000

239.0000	314.0000
239.0000	315.0000
239.0000	316.0000
239.0000	317.0000
239.0000	317.0000
238.0000	318.0000
238.0000	319.0000
238.0000	320.0000
238.0000	321.0000
238.0000	322.0000
238.0000	323.0000
238.0000	324.0000
238.0000	325.0000
238.0000	326.0000
238.0000	327.0000
238.0000	327.0000
237.0000	328.0000
237.0000	329.0000
237.0000	330.0000
237.0000	331.0000
237.0000	332.0000
237.0000	333.0000
237.0000	334.0000
237.0000	335.0000
237.0000	335.0000
236.0000	336.0000
237.0000	337.0000
237.0000	337.0000
237.0000	337.0000
236.0000	338.0000
236.0000	339.0000
236.0000	339.0000
235.0000	340.0000
235.0000	341.0000
235.0000	341.0000
234.0000	342.0000
234.0000	342.0000
233.0000	343.0000
233.0000	344.0000
233.0000	344.0000
232.0000	345.0000
232.0000	345.0000
231.0000	345.0000
230.0000	346.0000
230.0000	346.0000
229.0000	346.0000
228.0000	347.0000
228.0000	347.0000
227.0000	347.0000
226.0000	347.0000
225.0000	347.0000
224.0000	347.0000
223.0000	348.0000
223.0000	348.0000
222.0000	348.0000
221.0000	348.0000
220.0000	348.0000
219.0000	348.0000
218.0000	348.0000
217.0000	349.0000
217.0000	349.0000
216.0000	349.0000

215.0000	349.0000
214.0000	349.0000
213.0000	349.0000
212.0000	349.0000
211.0000	350.0000
210.0000	349.0000
209.0000	350.0000
209.0000	350.0000
208.0000	350.0000
207.0000	350.0000
206.0000	350.0000
205.0000	350.0000
204.0000	350.0000
203.0000	351.0000
203.0000	351.0000
202.0000	351.0000
201.0000	351.0000
200.0000	351.0000
199.0000	351.0000
198.0000	351.0000
197.0000	351.0000
196.0000	351.0000
195.0000	352.0000
195.0000	352.0000
194.0000	352.0000
193.0000	352.0000
192.0000	352.0000
191.0000	352.0000
190.0000	352.0000
189.0000	352.0000
188.0000	352.0000
187.0000	352.0000
186.0000	352.0000
185.0000	352.0000
184.0000	352.0000
183.0000	352.0000
182.0000	352.0000
181.0000	352.0000
181.0000	352.0000
180.0000	351.0000
179.0000	351.0000
178.0000	351.0000
177.0000	351.0000
177.0000	351.0000
176.0000	350.0000
175.0000	350.0000
174.0000	350.0000
174.0000	350.0000
173.0000	349.0000
172.0000	349.0000
172.0000	349.0000
171.0000	348.0000
170.0000	348.0000
170.0000	348.0000
169.0000	347.0000
168.0000	347.0000
168.0000	347.0000
167.0000	346.0000
167.0000	346.0000
166.0000	345.0000
165.0000	345.0000
165.0000	345.0000

164.0000	344.0000
164.0000	344.0000
163.0000	343.0000
163.0000	343.0000
162.0000	342.0000
162.0000	342.0000
161.0000	341.0000
161.0000	341.0000
160.0000	340.0000
160.0000	340.0000
159.0000	339.0000
159.0000	339.0000
158.0000	338.0000
158.0000	338.0000
158.0000	337.0000
157.0000	336.0000
157.0000	336.0000
156.0000	335.0000
156.0000	335.0000
156.0000	334.0000
155.0000	333.0000
155.0000	333.0000
155.0000	332.0000
154.0000	331.0000
154.0000	331.0000
154.0000	330.0000
153.0000	329.0000
153.0000	329.0000
153.0000	328.0000
153.0000	327.0000
152.0000	326.0000
152.0000	326.0000
152.0000	325.0000
151.9973	323.9999
150.9949	322.9997
150.9924	322.9996
150.9900	321.9995
150.9875	320.9994
150.9851	319.9993
149.9826	318.9992
149.9802	318.9990
149.9777	317.9989
149.9753	316.9988
149.9728	315.9987
149.9703	314.9986
148.9678	313.9984
148.9654	313.9983
148.9629	312.9982
148.9604	311.9981
148.9579	310.9979
148.9554	309.9978
148.9529	308.9977
147.9504	307.9976
147.9479	307.9975
147.9454	306.9973
147.9429	305.9972
147.9404	304.9971
147.9379	303.9970
147.9353	302.9968
147.9328	301.9967
147.9303	300.9966
147.9277	299.9965

146.9252	298.9963
146.9226	298.9962
146.9201	297.9961
146.9175	296.9960
146.9150	295.9958
146.9124	294.9957
146.9098	293.9956
145.9072	292.9955
145.9046	292.9953
145.9021	291.9952
145.8995	290.9951
145.8969	289.9950
145.8942	288.9948
145.8916	287.9947
145.8890	286.9946
145.8864	285.9945
144.8837	284.9943
144.8811	284.9942
144.8785	283.9941
144.8758	282.9939
144.8731	281.9938
144.8705	281.9937
145.8678	280.9935
145.8651	279.9934
145.8624	278.9933
145.8597	277.9932
145.8570	276.9930
145.8543	275.9929
145.8516	274.9928
145.8488	273.9926
145.8461	272.9925
145.8433	271.9924
145.8406	270.9922
145.8378	269.9921
145.8350	268.9919
145.8323	267.9918
145.8295	267.9917
146.8267	266.9915
146.8238	265.9914
146.8210	264.9913
146.8182	263.9911
146.8153	262.9910
146.8125	261.9908
146.8096	261.9907
147.8067	260.9906
147.8039	259.9904
147.8010	258.9903
147.7980	257.9901
147.7951	257.9900
148.7922	256.9899
148.7892	255.9897
148.7863	254.9896
148.7833	254.9894
149.7803	253.9893
149.7773	252.9891
149.7743	251.9890
149.7713	250.9888
149.7683	250.9887
150.7652	249.9885
150.7622	248.9884
150.7591	248.9882
151.7560	247.9881

151.7529	246.9879
151.7498	246.9878
152.7467	245.9876
152.7435	244.9875
152.7404	243.9873
152.7372	243.9872
153.7340	242.9870
153.7308	241.9869
153.7276	241.9867
154.7244	240.9865
154.7211	239.9864
154.7178	239.9862
155.7146	238.9861
155.7113	237.9859
155.7080	237.9857
156.7047	236.9856
156.7013	236.9854
157.6980	235.9853
157.6946	234.9851
157.6912	234.9849
158.6878	233.9848
158.6844	233.9846
159.6810	232.9844
159.6775	231.9843
159.6741	231.9841
160.6706	230.9839
160.6671	230.9837
161.6636	229.9836
161.6600	229.9834
162.6565	228.9832
162.6529	228.9831
163.6494	227.9829
163.6458	227.9827
164.6422	226.9825
164.6385	226.9824
165.6349	225.9822
165.6313	225.9820
166.6276	224.9818
166.6239	224.9816
167.6202	223.9815
167.6165	223.9813
168.6128	222.9811
168.6090	222.9809
169.6053	221.9807
169.6015	220.9805
169.5977	220.9804
170.5939	219.9802
170.5901	219.9800
171.5863	218.9798
171.5824	218.9796
172.5786	218.9794
173.5747	217.9792
173.5708	217.9790
174.5670	216.9789
174.5631	216.9787
175.5591	215.9785
175.5552	215.9783
176.5513	214.9781
176.5473	214.9779
177.5434	214.9777
178.5394	213.9775
178.5355	213.9773

179.5315	212.9771
179.5275	212.9769
180.5235	211.9767
180.5195	211.9765
181.5155	211.9763
182.5114	210.9761
182.5074	210.9760
183.5034	209.9758
183.4994	209.9756
184.4953	209.9754
185.4913	208.9752
185.4872	208.9750
186.4832	208.9748
187.4791	207.9746
187.4751	207.9744
188.4710	207.9742
189.4670	206.9740
189.4629	206.9738
190.4589	206.9736
191.4548	205.9734
191.4508	205.9732
192.4467	205.9730
193.4427	204.9728
193.4386	204.9726
194.4346	204.9724
195.4306	203.9722
195.4266	203.9720
196.4226	203.9718
197.4185	203.9716
198.4146	202.9714
198.4106	202.9712
199.4066	202.9710
200.4026	201.9708
200.3987	201.9706
201.3947	201.9704
202.3908	201.9703
203.3869	200.9701
203.3830	200.9699
204.3791	200.9697
205.3753	200.9695
206.3714	199.9693
206.3676	199.9691
207.3638	199.9689
208.3600	199.9688
209.3563	198.9686
209.3525	198.9684
210.3488	198.9682
211.3451	198.9680
212.3415	197.9678
212.3378	197.9677
213.3342	197.9675
214.3307	197.9673
215.3271	196.9671
215.3236	196.9670
216.3201	196.9668
217.3166	196.9666
218.3132	196.9665
219.3098	196.9663
220.3064	196.9661
221.3031	195.9660
221.2998	195.9658
222.2966	195.9657

223.2934	195.9655
224.2902	194.9653
224.2871	194.9652
225.2840	194.9650
225.2809	194.9649
226.2779	195.9647
227.2749	194.9646
227.2720	194.9645
228.2692	194.9643
229.2663	193.9642
229.2635	193.9640
230.2608	193.9639
231.2581	193.9638
232.2555	192.9637
232.2529	192.9635
233.2504	192.9634
233.2479	192.9633
234.2454	193.9632
235.2431	192.9630
235.2407	192.9629
236.2385	192.9628
237.2363	192.9627
238.2341	192.9626
239.2320	191.9625
239.2300	191.9624
240.2280	191.9623
241.2260	191.9622
242.2242	191.9621
243.2224	191.9620
244.2206	191.9619
245.2189	190.9619
245.2173	190.9618
246.2157	190.9617
247.2142	190.9616
248.2128	190.9616
249.2114	190.9615
250.2101	190.9614
251.2089	189.9614
251.2077	189.9613
252.2066	189.9613
253.2055	189.9612
254.2046	189.9612
255.2036	189.9611
256.2028	189.9611
257.2020	188.9610
257.2013	188.9610
258.2006	188.9610
259.2001	188.9609
260.1995	188.9609
261.1991	188.9609
262.1987	188.9609
263.1984	188.9609
264.1982	188.9609
265.1980	188.9608
266.1979	188.9608
267.2381	188.6178
268.9731	187.8275
269.4438	183.5837
268.6826	189.7603
269.2267	184.7378
268.8121	189.1811
268.5680	187.8702

```
268.0269  188.7254];
```
